# Identification of oxidative stress-associated biomarkers for inflammatory bowel disease through integrated machine learning and weighted gene co-expression network analysis

**DOI:** 10.3389/fimmu.2026.1763786

**Published:** 2026-06-10

**Authors:** Ruilong Kou, Yihao Liu, Chenyu Zhu, Tao Lu, Yu Chen, Zhiwei Qin, Bin Lan

**Affiliations:** 1 Department of Gastrointestinal Surgery 2 Section, The First Affiliated Hospital, Fujian Medical University, Fuzhou, China; 2 Department of Gastrointestinal Surgery, National Regional Medical Center, Binhai Campus of the First Affiliated Hospital, Fujian Medical University, Fuzhou, China; 3 Department of Gastrointestinal Surgery, The First Affiliated Hospital of Guilin Medical University, Guilin, China; 4 First Affiliated Hospital of Dalian Medical University, Dalian, China

**Keywords:** bioinformatic analysis, inflammatory bowel disease, machine learning, oxidative stress, WGCNA

## Abstract

**Background:**

Inflammatory bowel disease (IBD) is a complex chronic intestinal inflammatory disorder. Oxidative stress (OS) is crucial in the pathogenesis of IBD by promoting inflammation and disrupting the intestinal epithelial barrier.

**Methods:**

Weighted gene co-expression network analysis and machine learning were applied to publicly available gene expression data to identify OS-related biomarkers for IBD. Receiver operating characteristic curve, immune infiltration analysis, and single-cell analysis were used to evaluate diagnostic performance and cellular localization. RT-qPCR and Western blotting were conducted to validate gene expression.

**Results:**

Three hub genes (LCN2, APP, and TNFSF4) demonstrated strong diagnostic accuracy (AUC: 0.781, 0.805, and 0.754, respectively) in the discovery cohort (GSE3365). Furthermore, external validation in three independent cohorts also confirmed the robust diagnostic performance of the three hub genes. These genes were enriched in OS and inflammatory pathways, strongly correlated with infiltration of macrophages M0, monocytes, and neutrophils (p<0.01), and were highly expressed in goblet cells, stem cells, and T cells. DSS model validation confirmed significant upregulation at mRNA and protein levels.

**Conclusion:**

These OS-associated genes represent novel molecular targets for precise diagnosis and personalized treatment of IBD. This finding highlights the interplay among OS, immune dysregulation, and inflammation in IBD pathogenesis.

## 1 Introduction

Inflammatory bowel disease (IBD) refers to a multifaceted chronic intestinal inflammation, primarily encompassing Crohn’s disease (CD) and ulcerative colitis (UC) (1). Globally, the incidence rate of IBD climbed modestly from 4.22 per 100,000 in 1990 to 4.45 per 100,000 in 2021, reflecting a broader trend of increasing IBD cases worldwide (2). Clinically, IBD is characterized by recurrent episodes of chronic abdominal pain, weight loss, diarrhea, and extraintestinal manifestations (3). In addition to these physical symptoms, patients with IBD typically experience psychological comorbidities, including anxiety and depression, which further compound disease burden (4). Accordingly, understanding the pathogenesis of IBD is critical.

Oxidative stress (OS), an imbalance between reactive oxygen species (ROS) production and antioxidant defenses, represents a central pathogenic mechanism in IBD, driving epithelial barrier dysfunction, microbial dysbiosis, and immune dysregulation (5). At the epithelial level, OS compromises barrier integrity through coordinated disruption of multiple defense layers. OS downregulates tight junction proteins (Occludin, ZO-1), increasing paracellular permeability and facilitating bacterial translocation (6, 7). Concurrently, OS impairs the function of goblet cells and Paneth cells, diminishing mucus layer thickness and antimicrobial peptide secretion (8, 9). These epithelial defects compromise barrier integrity, which in turn promotes the dysbiosis of the gut microbiota (10). Additionally, OS disrupts the pathways of microbial metabolism, favoring the expansion of pathogenic bacteria while suppressing beneficial commensals (11), further exacerbating dysbiosis shifts. At the immune level, the resulting dysbiosis, coupled with persistent OS, activates pro-inflammatory signaling cascades, particularly the NF-κB and NLRP3 inflammasome pathways, leading to excessive immune activation and the overproduction of inflammatory cytokines (12, 13). These inflammatory mediators stimulate NADPH oxidase (NOX) in immune cells to generate additional ROS, creating a self-perpetuating ROS-inflammation circuit (14). Moreover, OS drives progressive immune dysregulation, particularly through disruption of the Treg/Th17 balance, ultimately perpetuating chronic intestinal inflammation in IBD (15).

OS-related biomarkers are regarded as significant indicators for assessing disease activity. Research has revealed that levels of OS-related biomarkers, such as malondialdehyde and total oxidant status, are considerably higher among patients experiencing IBD compared with those among healthy individuals in the control group, while levels of total antioxidant status among patients suffering from IBD are markedly reduced (16). These biomarkers can be utilized for IBD diagnosis and for monitoring treatment response (17). Despite their research potential, these OS biomarkers have not been adequately validated for routine clinical application in IBD (18). Furthermore, although existing commonly used biomarkers such as fecal calprotectin (FC) and C-reactive protein hold value in evaluating disease activity, they are not specific to IBD (19–21). Accordingly, detecting novel biomarkers and therapeutic targets is vital for promoting individualized medicine in IBD.

In recent years, high-throughput genomics technologies and advanced bioinformatics methods have been paving the way to address these limitations (22). Weighted gene co-expression network analysis (WGCNA) emerges as an effective tool for detecting gene modules and their associations in complex diseases, while machine learning (ML) algorithms offer sophisticated approaches for discovering and validating biomarkers (23–25). Recent studies have suggested that WGCNA combined with ML has been successfully applied to the discovery and validation of OS-related biomarkers in an assortment of diseases, comprising diabetic nephropathy and non-alcoholic fatty liver disease (26, 27). In terms of IBD, however, it remains to be explored how to apply WGCNA combined with ML to identify and validate OS-related biomarkers.

Based on the aforementioned evidence, three interconnected hypotheses are proposed: (i) patients with IBD harbor distinct OS-related gene signatures that can be systematically identified through integrated bioinformatics; (ii) these signatures demonstrate diagnostic performance and correlate with immune dysregulation; and (iii) they represent viable therapeutic targets for antioxidant-based and immunomodulatory interventions.

To test these hypotheses, this study aims to systematically identify and validate OS-associated biomarkers in IBD through a multi-stage analysis framework integrating WGCNA with dual ML algorithms (least absolute shrinkage and selection operator (LASSO) regression and Random Forest (RF)). Candidate biomarkers are subsequently validated through external cohort replication, single-cell RNA sequencing, immune infiltration profiling, and experimental verification via reverse transcription - quantitative polymerase chain reaction (RT-qPCR) and Western blotting (WB) in DSS-induced murine colitis models. It is anticipated that the panel of OS-related biomarkers detected in this research will offer a novel molecular tool for early detection, monitoring, and individualized treatment of IBD.

## 2 Methods

### 2.1 Data collection and data processing

The dataset GSE3365 (GPL96) was sourced from the Gene Expression Omnibus (GEO) database (https://www.ncbi.nlm.nih.gov/geo/), comprising 26 patients with UC, 59 patients with CD, and 42 healthy controls. Before analysis, quality control was performed: sample quality was assessed using the ‘goodSamplesGenes()’ function to detect missing values and anomalous samples, and hierarchical clustering was applied to identify potential outliers. All 127 samples met the quality control criteria and were retained for subsequent analysis. The expression data were normalized using the ‘Limma’ package (28). In addition, OS-related genes were obtained from the GeneCards database (https://www.genecards.org/), with a relevance score greater than 7 set as the selection criterion. Ultimately, 1,399 OS-related genes were identified. Meanwhile, the single-cell dataset GSE150115 (GPL18573) was acquired from the GEO database, consisting of tissue samples from 5 individuals suffering from UC. **Supplementary Table 1** illustrates the corresponding details. External validation was performed using three independent IBD datasets (GSE165512, GSE179285, and GSE75214) from the GEO database, encompassing 618 samples with varying disease subtype distributions (UC or CD proportions ranging from 24.7% or 75.3% to 56.4% or 43.6%). Notably, GSE75214 provided the stratification of disease activity (UC: 74 active and 23 inactive; CD: 59 active and 16 inactive), while GSE179285 documented inflammation status (UC: 23 inflamed and 32 non-inflamed; CD: 47 inflamed and 121 non-inflamed). All validation cohorts documented biopsy anatomical locations (ileum vs. colon), thereby supporting site-specific analysis. These cohorts included both microarray platforms (GPL6244 and GPL6480) and RNA-sequencing (GPL16791), allowing cross-platform validation of biomarker robustness. The characteristics of the cohorts, including sample size, platform specification, and disease subtype distribution, are detailed in **Supplementary Table 2**.

### 2.2 Detection of DEGs

Differentially expressed genes (DEGs) from a comparison of individuals with IBD and controls from the dataset GSE3365 were detected utilizing the ‘Limma’ package (28) in R (version 4.4.3). Specifically, low-expressed genes were filtered using the ‘filterByExpr’ function from the ‘edgeR’ package to retain genes with adequate expression levels. Data were normalized using the trimmed mean of M-values (TMM) method via ‘calcNormFactors’, followed by ‘voom’ transformation with quantile normalization (normalize = ‘quantile’) to stabilize variance across the expression range. Linear models were fitted using ‘lmFit’, and empirical Bayes moderation was applied via ‘eBayes’ to enhance statistical power. Genes were screened using the criteria of |log_2_FC|>0.5 and p<0.05. Those with log_2_FC>0.5 were designated ‘Up’, while those with log_2_FC<-0.5 were considered ‘Down’. The heatmap was generated with the ‘ComplexHeatmap’ package (29) from R, displaying the top 10 upregulated DEGs and the top 10 downregulated DEGs (ranked by log_2_FC). The volcano plot was created using the ‘ggplot2’ package (30) from R.

### 2.3 Establishment of weighted gene co-expression network and detection of pivotal module genes

A weighted gene co-expression network was constructed using the WGCNA package (31) from R to detect pivotal modules connected to the pathogenesis of IBD. Before WGCNA, gene expression data were log_2_-transformed (log_2_(x+1)). Genes were filtered based on median absolute deviation (MAD): only genes with MAD greater than the 25th percentile (or a minimum of 0.01) were retained to reduce computational burden while preserving biologically variable genes. Before WGCNA, sample clustering was implemented to pinpoint outliers. Soft-thresholding power (β) was selected when the scale-free topology fit index (R²) reached 0.825. With the minimum module size defined as 30 (minModuleSize=30), the dynamicTreeCut algorithm (deepSplit=2) was adopted to divide the modules, and the modules with similarity higher than 0.25 (mergeCutHeight=0.25) were merged. Networks were constructed using the ‘blockwiseModules’ function with Pearson correlation to calculate gene co-expression similarity. An unsigned topological overlap matrix (TOM) was computed to quantify network interconnectedness. Module eigengenes (MEs), defined as the first principal component of the expression matrix for each module, were calculated to represent overall module expression patterns. Pearson correlation was calculated between MEs and disease status (IBD vs. control) employing a binary design matrix. The module showing the strongest positive correlation with a significant correlation coefficient (p<0.05) was defined as the pivotal module, and the genes in the key module were extracted as the pivotal module genes.

### 2.4 Functional enrichment analysis of overlapping genes

The intersections among module genes, DEGs, and OS-related genes were identified and visualized using a 3-set Venn diagram. For the elucidation of the pathways and biological functions connected to the overlapping genes, enrichment analyses based on Kyoto Encyclopedia of Genes and Genomes (KEGG) and Gene Ontology (GO) were implemented using the ‘clusterProfiler’ package (32) from R. GO enrichment analysis encompasses three terms: molecular function, biological process, and cellular component. A p-value of <0.05 was set to screen enriched pathways. The ‘ggplot2’ package (version 3.5.1) from R was employed for the visualization of the results.

### 2.5 Network analysis of PPIs among overlapping genes

A protein-protein interaction (PPI) network of the overlapping genes was constructed based on the STRING database (version 12.0) (https://string-db.org/), with an interaction score threshold set at greater than 0.15. Visualization and analysis of the established network were conducted utilizing Cytoscape (version 3.10.3). Additionally, the CytoNCA plugin (version 2.1.6) for Cytoscape was employed to identify key gene nodes based on the Degree algorithm.

### 2.6 Identification of hub genes using ML algorithms

To detect core genes with optimal diagnosis and classification potential from the overlapping set, two independent ML algorithms were employed, comprising RF and LASSO regression. Firstly, LASSO regression was conducted with the ‘glmnet’ package (33) from R, where the optimal lambda value minimizing the mean squared error was determined via 10-fold cross-validation. Feature genes corresponding to non-zero coefficients were extracted in light of the minimum lambda value (lambda.min). Subsequently, an RF model was constructed utilizing the ‘randomForest’ package (version 4.7-1.2) from R with ntree=500. After the determination of the optimal tree count by analyzing the error rate curve, an optimized RF model was rebuilt. The importance of all genes was evaluated through the calculation of the mean decrease in Gini index (MeanDecreaseGini), and genes showing an importance score exceeding 3 were defined as pivotal feature genes selected by the RF algorithm. Ultimately, the intersection of these genes was determined as the ultimate hub gene set for subsequent validation and analysis.

### 2.7 Diagnostic performance of hub genes: ROC curve

Receiver operating characteristic (ROC) analysis was performed using the ‘ROCit’ package (version 2.1.2) and the ‘pROC’ package (version 1.18.5) (34) in R. The area under the ROC curve (AUC) value for each hub gene was calculated to evaluate the diagnostic performance of the identified hub genes in distinguishing patients experiencing IBD from individuals in the control group. Moreover, the diagnostic efficacy of the three genes was validated in the GSE165512, GSE179285, and GSE75214 datasets.

### 2.8 GSEA of hub genes

To further examine the biological functions and relevant pathways of the hub genes, a gene set enrichment analysis (GSEA) was implemented utilizing the ‘clusterProfiler’ package (32) from R, with KEGG pathways and GO terms as reference databases. Pathways with significant enrichment were identified according to the following criteria: nominal p-value <0.05, |NES|>1 (normalized enrichment score), and FDR (adjusted p-value) <0.25. The top 10 enriched pathways were singled out for visualization.

### 2.9 Construction of miRNA network

The ‘Funrich’ package (version 3.1.3) was adopted to identify potential miRNAs targeting the hub genes. The resulting miRNA-gene interaction network was visualized via Cytoscape (version 3.10.3).

### 2.10 Immune infiltration analysis

CIBERSORT, an algorithm for the deconvolution of gene expression, was employed to analyze the infiltration of immune cells from the GSE3365 dataset. The relative proportions of 22 types of immune cells in tissue samples were evaluated and visualized using stacked bar plots. In addition, differential infiltrations of these immune cells between individuals with IBD and controls were compared using the Wilcoxon rank-sum test and visualized through violin plots. The Spearman’s rank correlation analysis was conducted to appraise the associations between the expression of the key hub genes and the abundance of these immune cells, with results visualized through lollipop plots.

### 2.11 scRNA-seq data analysis

To additionally examine the expression patterns of OS-related hub genes across distinct immune cells among patients suffering from IBD, single-cell RNA sequencing (scRNA-seq) data analysis was carried out for the GSE150115 dataset using the ‘Seurat’ package (35).

First, low-quality cells were filtered by setting a minimum cell count of 3 and a minimum feature count of 200. Cells with fewer than 4,000 features and a mitochondrial gene percentage below 50% were retained to exclude potential dead cells and doublets.

Second, to reduce dimensionality and capture key sources of variation, 2,000 highly variable genes were detected through the approach of ‘variance stabilizing transformation (vst)’. Following gene scaling, a principal component analysis (PCA) was conducted to further decrease the dimension. The first 25 principal components were determined based on an ElbowPlot analysis of component contribution rates. For cluster analysis, cell clustering was conducted utilizing the Louvain algorithm (resolution=0.5) in light of the first 25 principal components. In order to visualize the clustering results, t-SNE and UMAP dimensionality reduction techniques were applied. During the clustering, marker genes identified for individual cell clusters were determined utilizing the FindAllMarkers function, and particular attention was paid to genes showing an average log_2_ fold change (avg_log_2_FC) above 1. Various types of cells were then annotated by detecting marker genes in different clusters with reference to the CellMarker database (http://bio-bigdata.hrbmu.edu.cn/CellMarker/). Third, the expression of the selected hub genes across various types of cells was visualized utilizing the FeaturePlot.

### 2.12 Pseudotime trajectory, cell-cell communication, and virtual knockout simulation analysis

To comprehensively elucidate the mechanisms of hub genes in the IBD microenvironment, three complementary in-depth analyses were performed to investigate their temporal dynamics, spatial interactions, and functional consequences.

#### 2.12.1 Pseudotime trajectory analysis

Pseudotime trajectory analysis was performed using Monocle 2 (version 2.34.0) to investigate dynamic expression patterns during the differentiation of intestinal epithelial cells. Given the high expression of LCN2 and APP in stem cells and goblet cells, these two types of cells were extracted from the Seurat object for reconstructing differentiation trajectories. A CellDataSet object was created with a negative binomial distribution (expressionFamily = negbinomial.size, lower detection limit = 0.5). Trajectory-dependent genes were identified using ‘differentialGeneTest’ with a likelihood ratio test comparing the full model (~Celltype + orig.ident) against the reduced model (~orig.ident) to account for batch effects. Genes with a q-value < 0.01 were selected as ordering genes. Dimensionality reduction was performed with batch correction (residualModelFormulaStr = ‘~orig.ident’), and cells were ordered with ‘orderCells’.

#### 2.12.2 Cell-cell communication analysis

Cell-cell communication analysis was conducted using CellChat (version 1.6.1) with an expression-based cell subtyping strategy. Stem cells and goblet cells were subdivided based on LCN2 and APP expression (log-normalized data, expression > 0 defined as positive), generating four subgroups per gene: stem_cells_gene(+/−) and goblet_cells_gene(+/−). For TNFSF4, T cells were similarly subdivided. CellChat analysis was performed based on the human ligand-receptor database (CellChatDB.human). Overexpressed ligands and receptors were identified, and expression values were projected onto protein-protein interaction networks (PPI.human). Communication probabilities were calculated with raw expression values and population size correction (raw.use = TRUE, population.size = TRUE), filtering interactions involving fewer than 10 cells. Pathway-level networks were inferred using ‘computeCommunProbPathway’, and network centrality was quantified with ‘netAnalysis_computeCentrality’.

#### 2.12.3 Virtual knockout simulation

In silico gene knockout simulations were performed using scTenifoldKnk (version 1.0.2) to predict functional consequences of hub gene perturbation. The top 3,000 highly variable genes were extracted from raw count data as input. For each hub gene (APP, LCN2, or TNFSF4), virtual knockout was simulated with quality control parameters: mitochondrial gene percentage < 10% and minimum library size > 1,000 genes. Gene regulation networks were constructed using 10 subnetworks (nc_nNet = 10), each based on 500 randomly sampled cells (nc_nCells = 500) with three principal components (nc_nComp = 3). Differential regulation was assessed by comparing wild-type and knockout networks. Genes with |Z-score| > 2 and adjusted p-value < 0.05 were considered significantly affected. The results were visualized through bar plots (top 10 genes ranked by |FC|) and volcano plots (Z-score vs. -log_10_ (p.adj).

### 2.13 Experimental validation

#### 2.13.1 Animal model establishment and sample collection

Male C57BL/6 mice (aged 6–8 weeks old; weighing 18–22 g) were sourced from Hangzhou Ziyuan Laboratory Animal Technology Co., Ltd. Each animal experiment was undertaken under the approval of the Institutional Animal Ethics Committee of the First Affiliated Hospital of Fujian Medical University (Approval No.: [IACUC FJMU 2023-0259]) and complied with the institutional guidelines.

After 7 days of acclimatization under standard conditions, these mice were randomly divided into two groups (n = 3 per group): a blank control group and a model group. In the model group, colitis was triggered by the administration of freshly prepared 3% (w/v) dextran sulfate sodium (DSS) (MW 36–50 kDa) in drinking water for 7 days, while the control group was administered normal drinking water. The disease activity index (DAI) was monitored and computed daily.

On the eighth day, these mice were humanely euthanized via cervical dislocation. Colon tissues were immediately harvested and measured for length. Fresh colon tissues were taken for flow cytometry analysis. Proximal colon segments were flash-frozen in liquid nitrogen and maintained in a -80 °C freezer for extracting RNAs and proteins. Mid-colon segments were fixed in 4% paraformaldehyde for histological examination (HE).

#### 2.13.2 HE

Colon tissue samples were fixed in 4% paraformaldehyde and processed for histology at our institutional pathology laboratory according to standard protocols. Briefly, fixed tissues underwent dehydration, paraffin embedding, sectioning (5 μm thickness), and H&E staining according to the laboratory’s standard operating procedures (SOP). Stained sections were examined under a light microscope (Nikon ECLIPSE CI-L, Japan; Nikon ECLIPSE 80I for high-resolution imaging, Japan) at multiple magnifications. Whole slide imaging was performed using a PANNORAMIC MIDI/SCAN 150 digital slide scanner (3DHISTECH, Hungary). Experienced pathologists performed the histopathological evaluation, assessing basic pathological changes including basic pathological changes including congestion, edema, degeneration, hyperplasia, fibrosis, calcification, metaplasia, necrosis, and inflammatory infiltration. Moreover, representative areas were photographed.

#### 2.13.3 RT-qPCR

Total RNA was isolated from frozen colon tissues utilizing the TransZol Up Plus RNA Kit (ER501-01, TRANS), referring to the instructions. The purity and concentration of RNA were measured with a UV spectrophotometer. Genomic DNA removal and cDNA synthesis were conducted using the TransScript^®^ Uni All-in-One First-Strand cDNA Synthesis SuperMix for qPCR (AU341, TRANS). RT-qPCR was implemented with MagicSYBR Mixture (CW3008, Cowin Biotech) on a CFX Connect™ Real-Time PCR Detection System (Bio-Rad Laboratories (Shanghai) Co., Ltd.). Reaction mixtures were prepared according to the manufacturer’s instructions, and a standard three-step PCR protocol was performed: pre-denaturation at 95 °C, followed by 40 cycles of denaturation, annealing, and extension. Expression levels of genes were standardized utilizing the internal reference gene GAPDH as the benchmark, and relative levels of expression were determined using the method of 2^(-ΔΔCt). Sangon Biotech (Shanghai) Co., Ltd. synthesized all primers, and the sequences are presented in **Supplementary Table 3**.

#### 2.13.4 WB analysis

Total protein was isolated from frozen colon tissues utilizing RIPA lysis buffer. The concentration of protein was identified with a BCA protein quantification kit. Equivalent amounts of protein (30 μg) and protein marker were resolved by 10% SDS-PAGE and then transferred onto a PVDF membrane. Following the transfer, the membrane was blocked with 3% skim milk for 1 hour at room temperature and was then incubated overnight at 4 °C with the primary antibodies (diluted with Beyotime primary antibody dilution buffer): rabbit anti-TNFSF4 antibody (1:4000; 82794-7, Proteintech), rabbit anti-APP antibody (1:800; 27320-1, Proteintech), rabbit anti-LCN2 antibody (1:5000; 26991-1, Proteintech), and mouse anti-β-actin antibody (1:50000; 66009-1-lg, Proteintech) as the loading controls. Washed using TBST, the membrane was then incubated for 2 hours at room temperature with HRP-conjugated secondary antibodies (diluted with Beyotime secondary antibody dilution buffer): HRP-goat anti-rabbit IgG (1:10000; RGAM001, Proteintech) for TNFSF4, APP, and LCN2, and HRP-goat anti-mouse IgG (1:10000; RGAR001, Proteintech) for β-actin. The membrane was washed again using TBST. The PVDF membrane was then incubated with ultrasensitive luminescence solution, and chemiluminescent signals were detected using a fully automated chemiluminescence imaging analysis system. Protein band intensities were analyzed via ImageJ, and expression levels of protein were standardized using β-actin as a reference.

### 2.14 Statistical analysis

All statistical analyses were performed using R and GraphPad Prism (version 10.1.2). Differences between the two groups were assessed using the Mann-Whitney U test (for data without a normal distribution) or the Student’s t-test (for data with a normal distribution). The predictive performance of the candidate genes was assessed via ROC curves. A statistically significant difference was determined as p <0.05. All experiments were implemented with at least 3 independent biological replicates.

## 3 Results

The analytical framework of this research is presented in **Supplementary Figure 1**, which systematically outlines the progression from biomarker discovery through experimental validation.

### 3.1 Detection of IBD_DEGs

**Figure 1:**
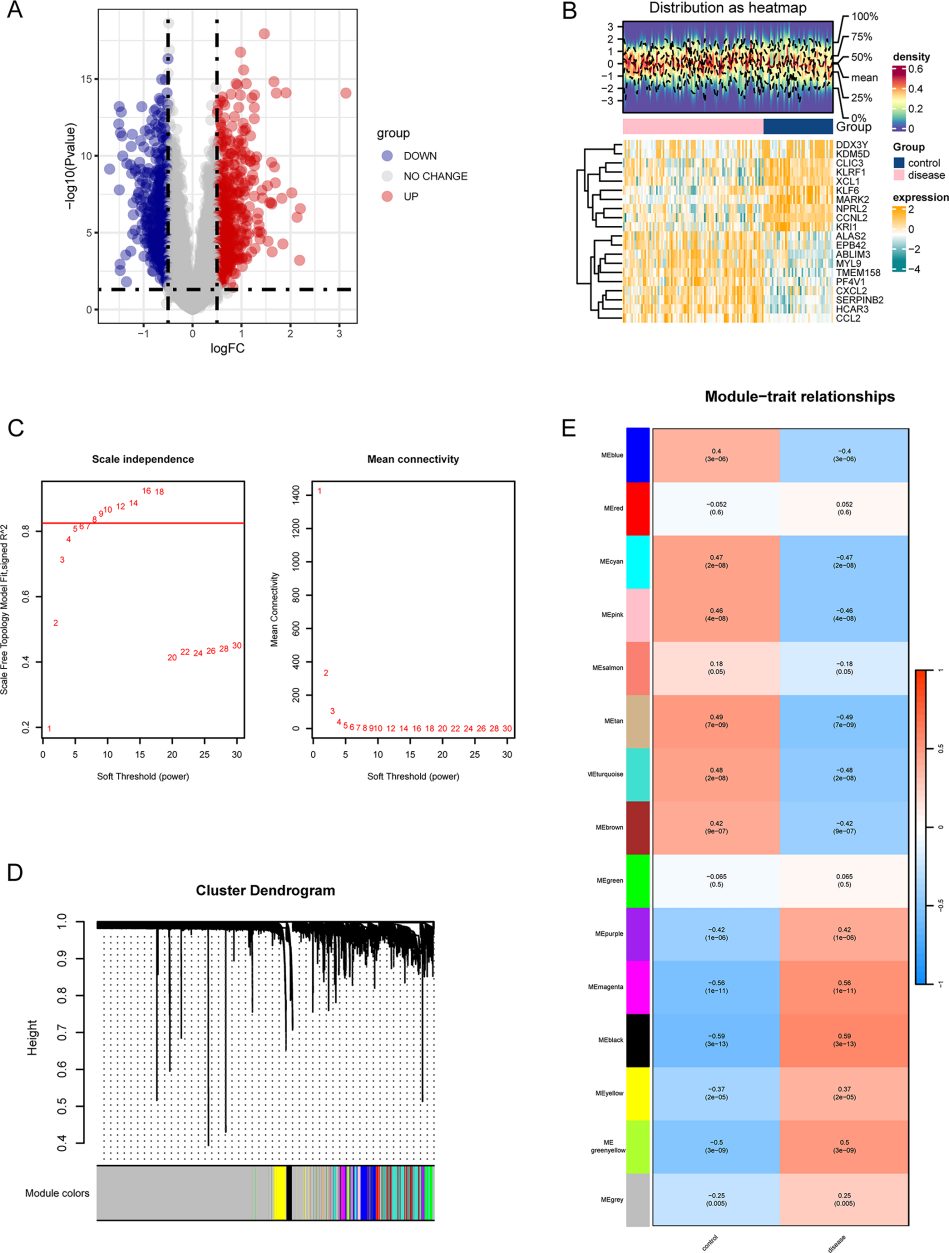
WGCNA and detection of DEGs. **(A)** Volcano plot for DEGs from the GSE3365 dataset: dots in red represent upregulated genes, blue dots represent downregulated genes, and grey dots indicate non-significantly differentially expressed genes. **(B)** Heatmap for DEGs in the GSE3365 dataset: the upper section displays the expression density heatmap, and the lower section shows the expression density distribution. **(C)** Plot for Soft Thresholding Power Selection (scale-free topology): the left plot indicates the scale-free model fit index, and the right plot shows the mean connectivity. **(D)** Gene dendrogram and modules at β = 9. **(E)** Module-IBD correlation heatmap: red suggests positive correlation; blue stands for negative correlation.

To detect genes potentially implicated in IBD pathogenesis, the analysis of differential expression was conducted between IBD samples (n = 85) and control samples (n = 42) from the GSE3365 dataset. Based on the filtering criteria: p < 0.05 and |log_2_FC| > 0.5, a total of 959 DEGs in total were identified, comprising 489 upregulated DEGs and 470 downregulated ones (**Figure 1A**). The distribution of the DEGs was visualized in a volcano plot (**Figure 1A**). A hierarchical clustering heatmap analysis was conducted for the top 20 most significant genes that were differentially expressed (ranked by |log_2_FC| value, 10 upregulated genes and 10 downregulated ones), and the analysis revealed distinct expression patterns between the controls and patients with IBD (**Figure 1B**).

### 3.2 Identification of IBD-associated gene modules via WGCNA

To identify the gene co-expression network related to IBD, a WGCNA was carried out on the GSE3365 dataset. Sample clustering showed that no outlier samples needed to be removed. Analysis of network topology across different soft thresholds indicated that β = 9 was the most appropriate soft threshold, reaching a scale-free topology fit index of 0.854 while maintaining sufficient mean connectivity (**Figure 1C**). Using this threshold, 15 distinct gene modules were identified through cluster analysis (**Figure 1D**). Correlation analysis between module eigengenes and IBD status revealed that the black module had the strongest positive association with IBD status (r = 0.59, p <3e-13) (**Figure 1E**). Ultimately, 167 genes in total were extracted from the black module.

### 3.3 Identification of overlapping genes from DEGs, pivotal module genes, and OS-associated genes

**Figure 2:**
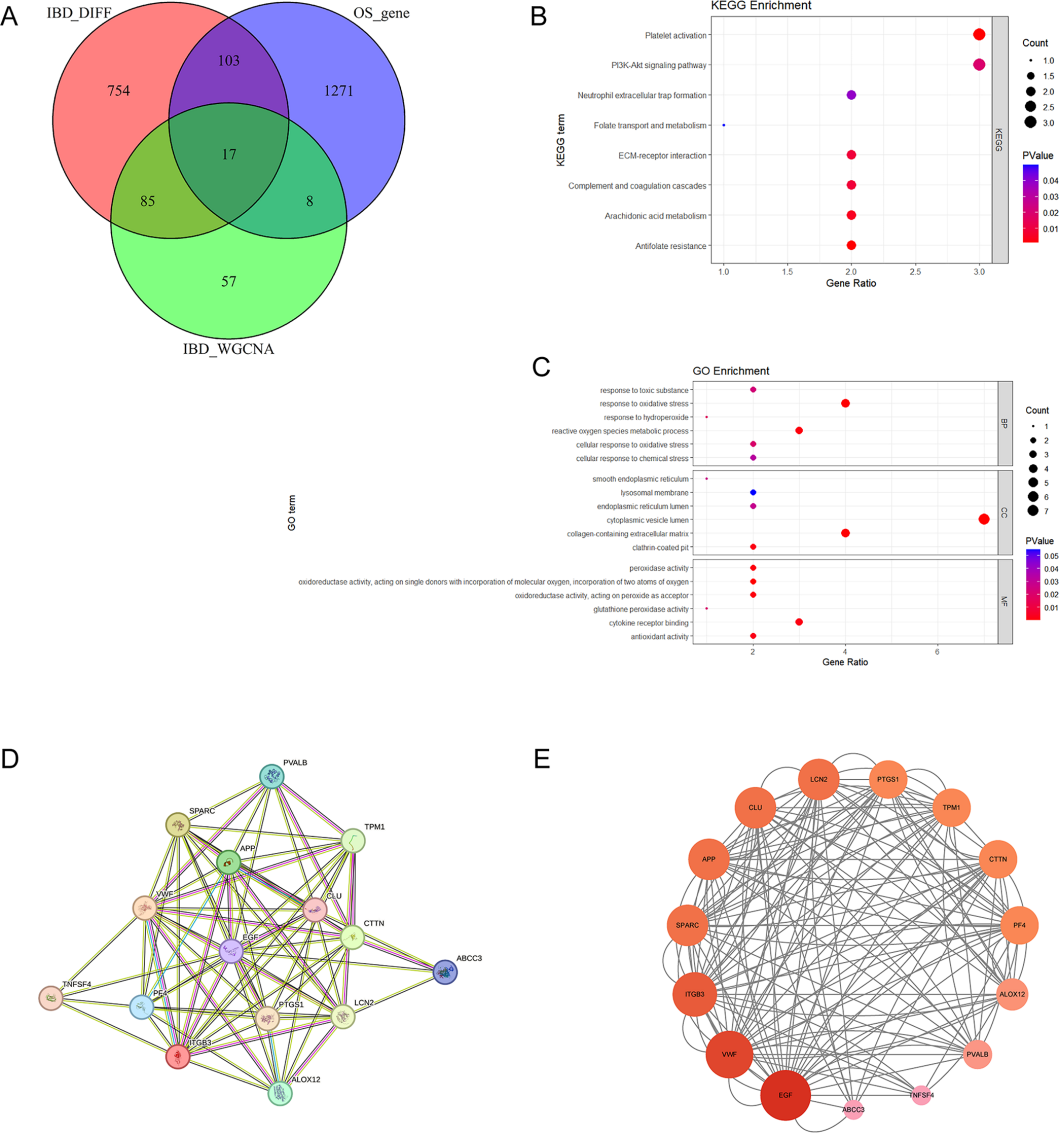
Screening of overlapping DEGs, functional enrichment, and PPI analysis. **(A)** Venn diagram for IBD_DIFF, OS-related genes, and genes identified by IBD_WGCNA. **(B)** Bubble plot for enrichment analysis based on KEGG of the 17 overlapping genes. **(C)** Bubble plot for enrichment analysis based on GO of the 17 overlapping genes. Functions of the overlapping genes are illustrated from 3 dimensions: molecular function, biological process, and cellular component. **(D)** PPI network of the 17 overlapping genes (isolated nodes were removed). **(E)** Importance ranking of the 17 overlapping genes.

The intersection of DEGs from the GSE3365 dataset, pivotal module genes from the WGCNA, and 1399 OS-associated genes acquired from the GeneCards database yielded 17 genes, which were designated as the overlapping genes of IBD-associated OS (**Figure 2A**).

### 3.4 Functional enrichment analysis and network analysis of PPIs among overlapping genes

To clarify the potential biological functions and metabolism pathways of the 17 overlapping OS−associated genes in IBD, KEGG and GO enrichment analyses were performed. KEGG pathway analysis manifested that the 17 genes predominantly participated in pathways closely associated with IBD pathogenesis, comprising ‘PI3K-Akt signaling pathway’, ‘ECM-receptor interaction’, ‘formation of neutrophil extracellular traps (NETs)’, ‘platelet activation’, and ‘arachidonic acid metabolism’ (**Figure 2B**). GO enrichment analysis demonstrated marked enrichment in biological processes, such as response to OS, cellular response to OS, and metabolic process of ROS. For cellular component, notable enrichment was observed in the cytoplasmic vesicle lumen, collagen-containing extracellular matrix, and endoplasmic reticulum. Regarding molecular function, significant enrichment was identified in antioxidant activity, glutathione peroxidase activity, and oxidoreductase activity (**Figure 2C**). These results collectively suggested that the final set of 17 overlapping genes might be crucial in the processes of OS in IBD.

To investigate the interplay among the 17 overlapping genes, a PPI network was constructed based on the STRING database (**Figure 2D**). The ‘CytoNCA’ plugin (version 2.1.6) for the Cytoscape (version 3.10.3) was further employed to identify relevant gene nodes based on the Degree algorithm, indicating their potential importance within the network (**Figure 2E**).

### 3.5 Identification of hub genes using ML algorithms

**Figure 3:**
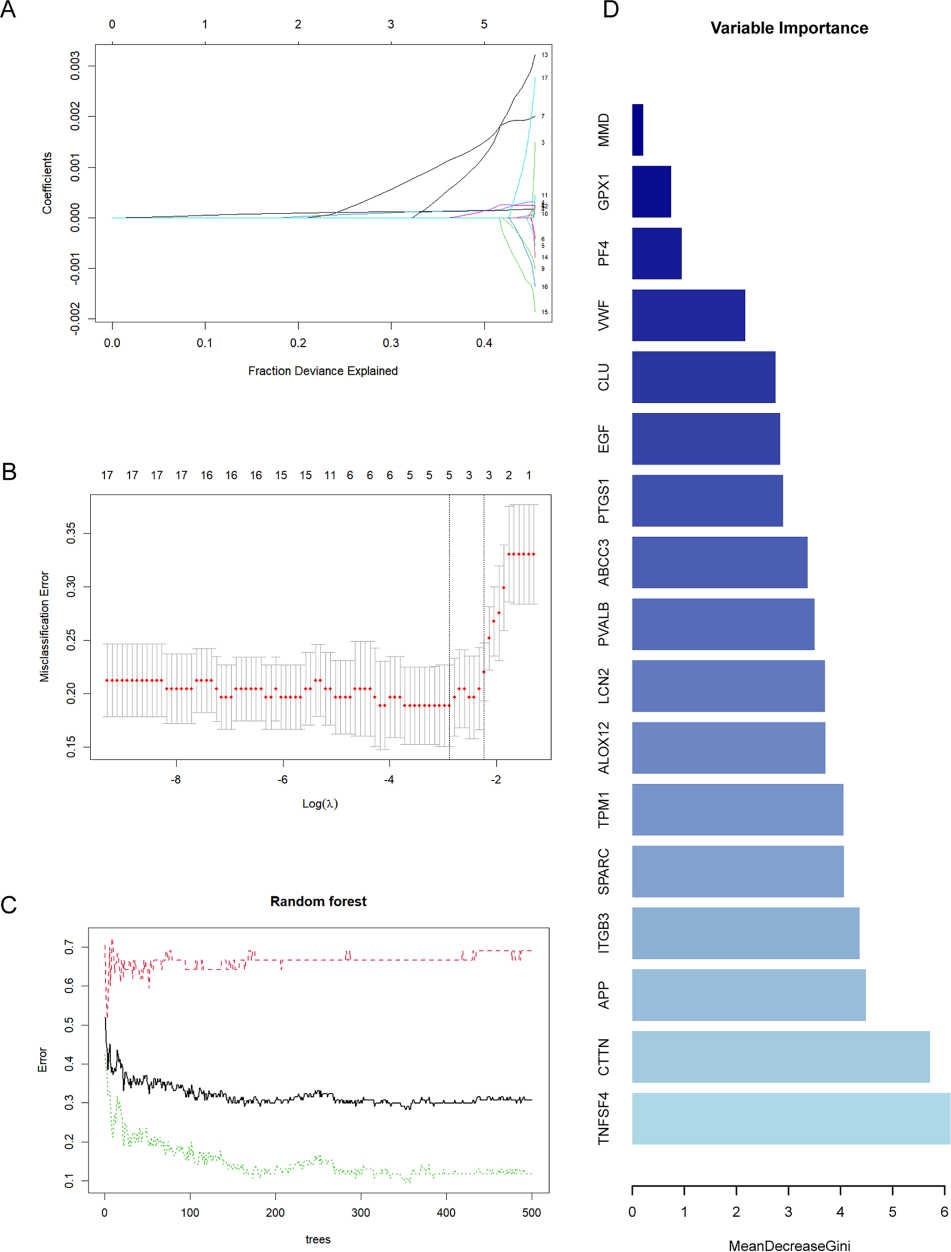
Identification of hub genes using ML algorithms. **(A)** Diagram for LASSO regression path. **(B)** Cross-validation curve for LASSO regression. **(C)** Random forest classification error curve. **(D)** Ranking of variable importance.

Based on the optimal lambda value (lambda.min = 0.05621942) from LASSO analysis, five genes were identified: LCN2, APP, MMD, TNFSF4, and GPX1 (**Figures 3A, B**).

Through the RF algorithm, genes with an importance score exceeding 3 were designated as key genes. As a result, 11 genes were identified, comprising LCN2, APP, TNFSF4, CTTN, ITGB3, SPARC, TPM1, ALOX12, PVALB, ABCC3, and PTGS1 (**Figures 3C, D**). Finally, through the intersection of genes detected by these two algorithms, 3 hub genes were obtained, including LCN2, APP, and TNFSF4.

### 3.6 Diagnostic performance and external validation of hub genes

**Figure 4:**
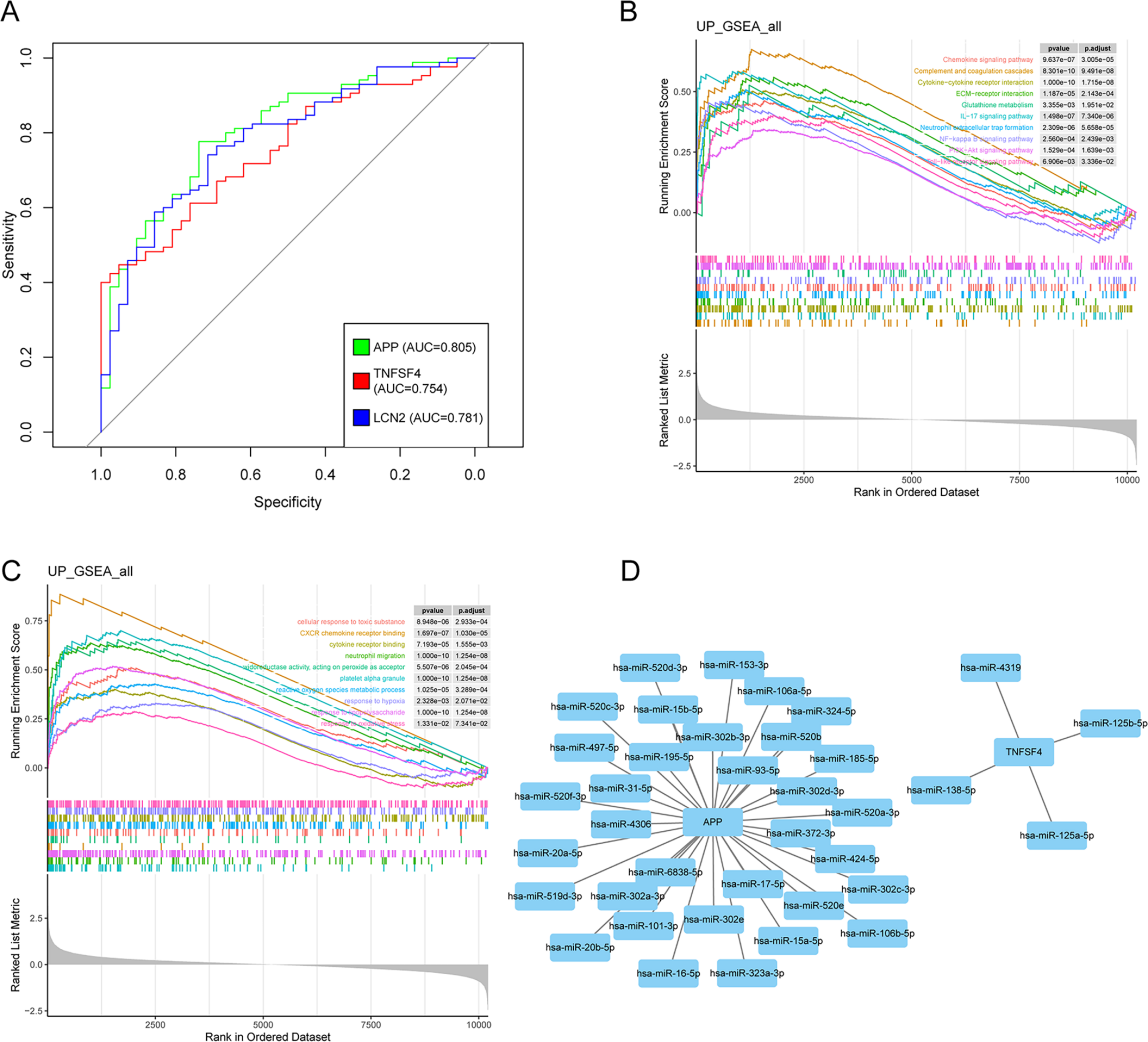
Diagnostic performance and functional enrichment analysis of hub genes. **(A)** ROC curve for the 3 hub genes (APP, TNFSF4, and LCN2) in GSE3365. **(B)** GSEA (KEGG pathway). **(C)** GSEA (GO biological process). **(D)** Interaction network of miRNAs with hub genes.

The 3 hub genes were evaluated using ROC curve analysis in the discovery cohort (GSE3365), revealing that all exhibited AUC values >0.7 and demonstrated favorable predictive accuracy in distinguishing IBD from control samples (**Figure 4A**). APP showed the highest diagnostic accuracy (AUC = 0.805), followed by LCN2 (AUC = 0.781) and TNFSF4 (AUC = 0.754). To validate these findings and assess potential overfitting, external validation in the three independent IBD cohorts (GSE75214, GSE179285, and GSE165512) was performed. These cohorts comprised a total of 618 samples across diverse populations and platforms (**Supplementary Table 2**; **Supplementary Figure 2**). The validation revealed that LCN2 demonstrated exceptional diagnostic performance, achieving AUC values of 0.953 (GSE75214) and 0.844 (GSE179285), which matched or exceeded the performance observed in the discovery cohort. Even in the most conservative cohort (GSE165512), LCN2 maintained modest but detectable performance (AUC = 0.643). APP and TNFSF4 demonstrated moderate yet consistent diagnostic value across all validation cohorts, with AUC ranges of 0.606-0.677 and 0.632-0.686, respectively. The consistent performance of APP and TNFSF4 across all independent datasets (all AUC >0.60), despite being lower than in the discovery cohort, confirmed their genuine diagnostic potential.

These results indicated that these 3 hub genes identified through ML algorithms hold potential as diagnostic biomarkers for IBD and may provide novel molecular targets for an early identification and a precise treatment of IBD.

### 3.7 GSEA of hub genes

GSEA was carried out to identify pathways and biological functions connected to the hub genes, and the 10 pathways that were most significantly enriched were visualized. The results of KEGG pathway analysis are demonstrated in **Figure 4B**. Multiple pathways associated with OS, immune response, and inflammation were markedly enriched in IBD samples, including ‘Chemokine Signaling Pathway’, ‘NF-kappa B Signaling Pathway’, ‘IL-17 Signaling Pathway’, ‘Glutathione Metabolism’, ‘Cytokine-Cytokine Receptor Interaction’, and ‘PI3K-Akt Signaling Pathway’.

The GO functional enrichment analysis is detailed in **Figure 4C**. The analysis revealed significant enrichment of biological processes associated with redox processes and inflammatory responses in IBD samples, such as ‘metabolic process of ROS’, ‘neutrophil migration’, ‘response to lipopolysaccharide’, ‘CXCR chemokine receptor binding’, ‘oxidoreductase activity, acting on peroxide as acceptor’, ‘cytokine receptor binding’, and ‘response to OS’.

These results have highlighted the critical roles of the identified hub genes in inflammatory responses, OS regulation, and processes of cellular metabolism within the context of IBD pathogenesis.

### 3.8 miRNA regulation network of hub genes

To investigate the mechanisms of potential post-transcriptional regulation, the ‘FunRich’ package was utilized to forecast miRNAs targeting the hub genes, and their regulation network of miRNAs was successfully established (**Figure 4D**). Among the hub genes in the regulation network of miRNAs, APP showed the highest number of interactions, followed by TNFSF4, suggesting their potential roles as major regulators in the OS response of IBD. Notably, no miRNA regulators of LCN2 were identified in the database.

### 3.9 Immune infiltration analysis

**Figure 5:**
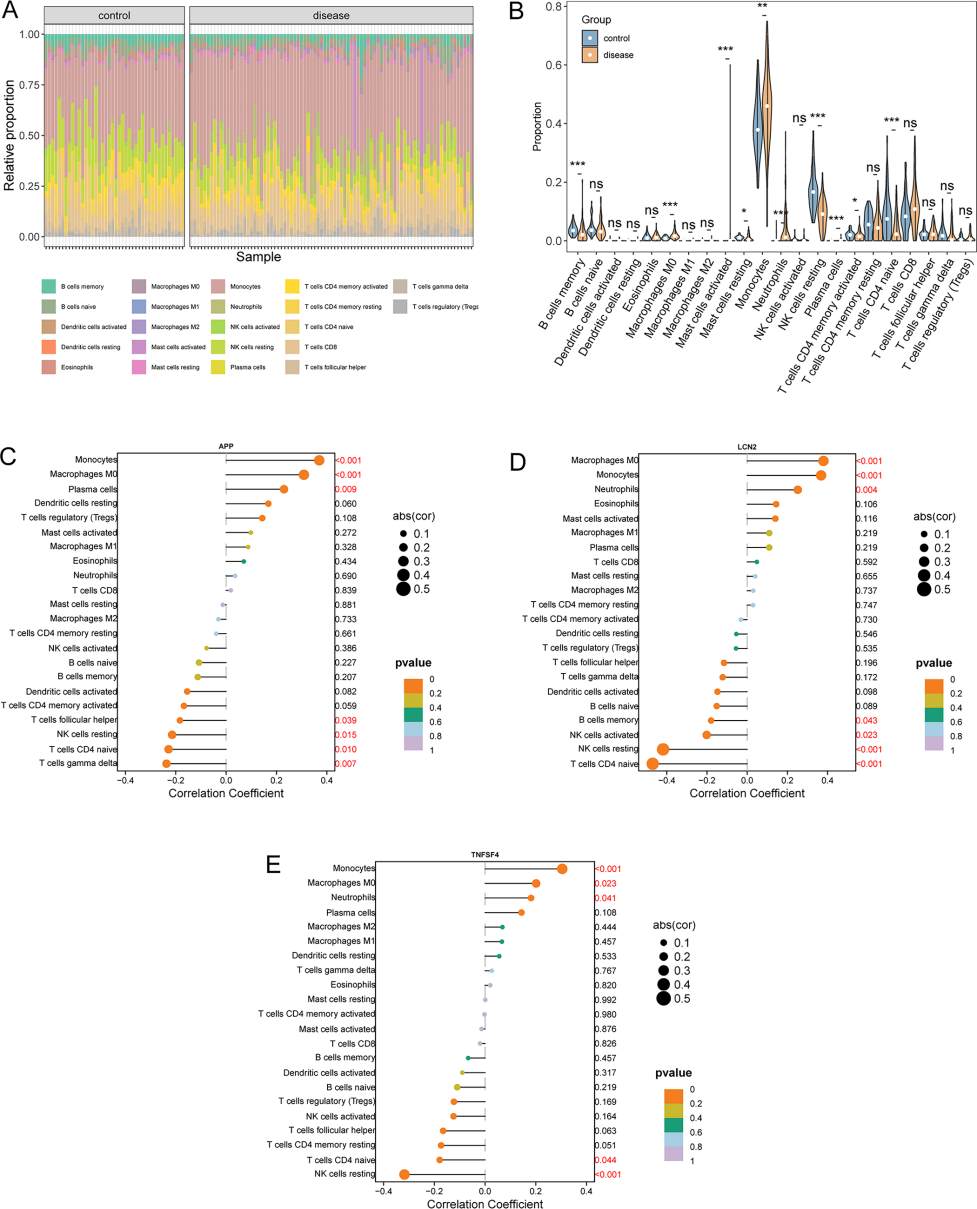
Analysis of correlation between infiltration of immune cells and hub genes. **(A)** Stacked bar plot: the relative proportion distribution of 22 kinds of immune cells in the samples of the control group and those of the IBD group. **(B)** Violin plot for differential analysis of immune cells: comparison of infiltration levels of distinct immune cells between the control group and the IBD group (*P < 0.05, **P < 0.01, ***P < 0.001; ns, no significant difference). **(C–E)** Lollipop charts for correlations of APP, LCN2, and TNFSF4 with different immune cells, respectively.

The LM22 feature gene matrix from the CIBERSORT package (containing signature genes for 22 types of immune cells) was used to estimate the proportions of immune cells based on the expression profiles of the GSE3365 dataset. A stacked bar plot displayed the relative ratios of distinct immune cells in an individual tissue sample (**Figure 5A**). The results manifested that the ratios of numerous immune cells in the IBD group significantly increased, including innate immune cells like Macrophages M0, Neutrophils, and Monocytes.

A violin plot was used to compare the abundance differences of various immune cells between the IBD group and the control group (**Figure 5B**). To highlight statistical differences, the Wilcoxon rank-sum test was applied for intergroup comparisons, with significance levels demonstrated by asterisks (*p < 0.05, **p < 0.01, ***p < 0.001). The results demonstrated marked differences in multiple kinds of immune cells between patients suffering from IBD and healthy individuals in the control group. Immune cell populations with significantly increased infiltration (p <0.01) in the IBD group included Macrophages M0, Neutrophils, and Monocytes, while significantly decreased immune cell populations encompassed memory B cells and naive CD4 T cells.

Furthermore, the association between infiltration levels of various immune cells and the hub genes was analyzed (**Figures 5C–E**). The Spearman’s rank correlation analysis was used to assess the association. The results manifested that APP expression demonstrated a considerable positive correlation with Monocytes, Macrophages M0, and Plasma cells, while considerably negatively associated with resting NK cells, T follicular helper cells, γδ T cells, and naive CD4 T cells (**Figure 5C**). LCN2 expression indicated a marked positive correlation with Monocytes, Neutrophils, and Macrophages M0, while considerably negatively correlated with resting NK cells, activated NK cells, naive CD4 T cells, and memory B cells (**Figure 5D**). TNFSF4 was significantly positively correlated with Monocytes, Macrophages M0, and Neutrophils, while showing a significant negative correlation with resting NK cells and naive CD4 T cells (**Figure 5E**). These findings have suggested that OS-related hub genes of IBD may participate in the occurrence and progression of IBD by modulating the immune microenvironment.

### 3.10 Expression patterns of hub genes at a single-cell level

**Figure 6:**
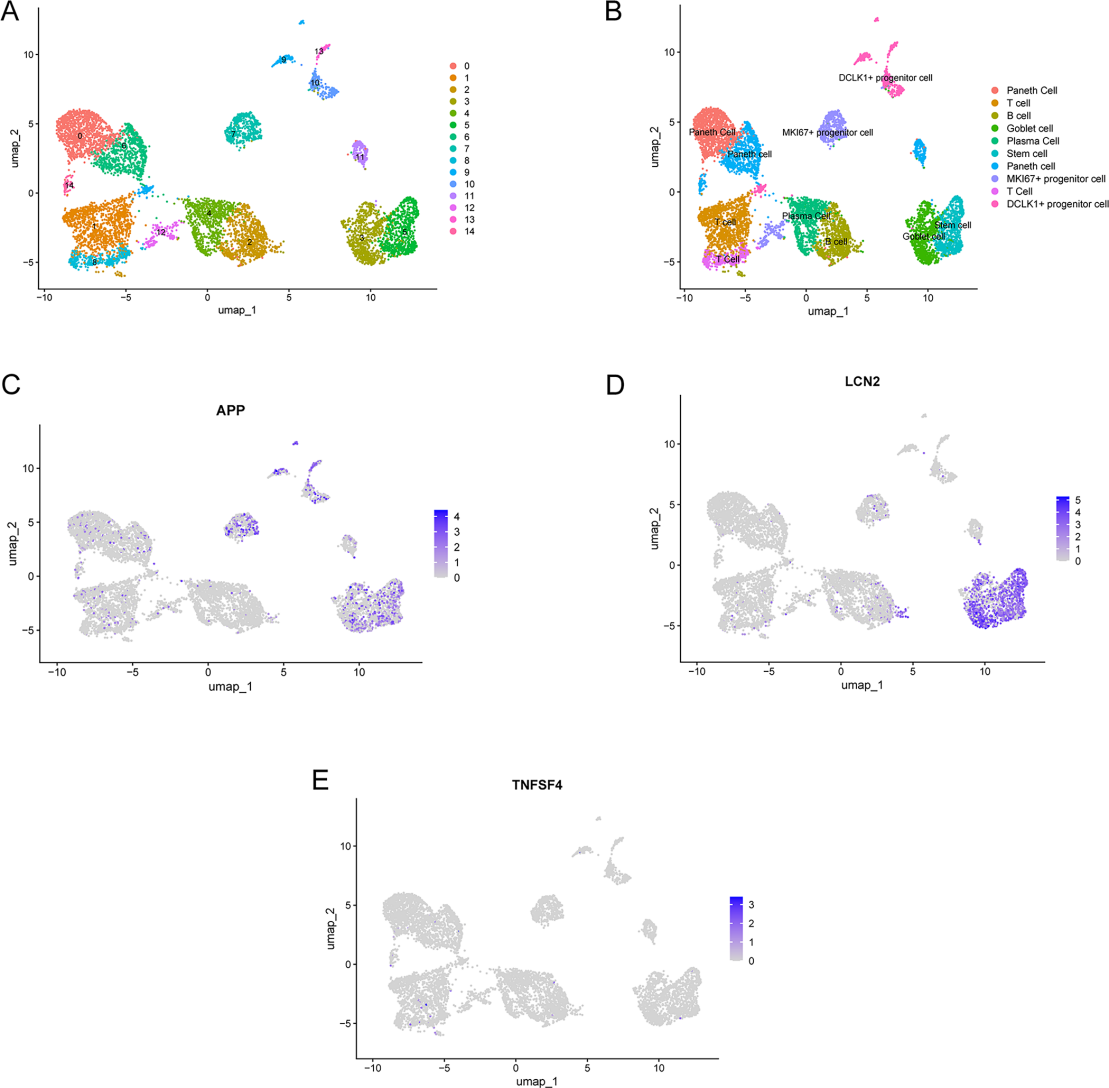
UMAP clustering analysis and expression distribution of hub genes at a single-cell level. **(A)** UMAP clustering plot (full cell populations): cells were divided into 15 major clusters based on their expression profiles, with each cluster marked by a distinct color. **(B)** UMAP plot annotated by type of cell. **(C–E)** UMAP distribution of APP, LCN2, and TNFSF4 expression: color gradient from grey to blue indicates the level of expression of the hub genes from low to high.

For deeper insight into the cellular distribution of hub genes in IBD, scRNA-seq data analysis was performed for the dataset GSE150115 from patients with UC. Cells were visualized using UMAP nonlinear dimensionality reduction and clustered via the Louvain algorithm (resolution = 0.5). A total of 15 distinct cell clusters were determined (**Figure 6A**). From the expression of canonical marker genes, these clusters were annotated as eight cell types: Paneth cells (clusters 0, 6, 11, 14), T cells (clusters 1, 8), B cells (cluster 2), plasma cells (cluster 4), stem cells (cluster 5), goblet cells (cluster 3), and MKI67^+^ (clusters 7, 12) and DCLK1^+^ progenitor cells (clusters 9, 10, 13) (**Figure 6B**).

The expression of the 3 hub genes, comprising APP, LCN2, and TNFSF4, was further examined across different cell types (**Figures 6C, E**). The examination showed that APP was expressed in multiple types of cells, with higher levels observed in goblet cells, stem cells, and MKI67^+^ and DCLK1^+^ progenitor cells (**Figure 6C**). LCN2 was also broadly expressed across an assortment of cells, with prominent expression in goblet cells and stem cells (**Figure 6D**). In contrast, TNFSF4 was primarily and specifically expressed in T cells (**Figure 6E**).

### 3.11 Dynamic expression patterns of hub genes along a cellular differentiation trajectory

To investigate the temporal dynamics of hub genes during intestinal epithelial cell differentiation, a pseudotime trajectory analysis was performed on stem cells and goblet cells extracted from the GSE150115 dataset using Monocle 2. The reconstructed trajectory exhibited a bifurcating structure spanning 0–15 units across the pseudotime sequence, with cells being partitioned into five distinct states (**Supplementary Figure 3A**). Stem cells predominantly occupied early pseudotime positions on the left and lower branches, whereas goblet cells were enriched in the later pseudotime regions of the right branch. The central decision node (State 2) represented a critical bifurcation point within the differentiation continuum.

The differential gene expression analysis identified the top 50 quasi-chrono-dependent genes (ranked by q-value), which were clustered into two distinct temporal patterns (**Supplementary Figure 3B**). Cluster 1 (33 genes) exhibited high expression early on, which gradually decreased. This cluster included epithelial markers (KRT19, KRT20, EPCAM), cell adhesion molecules (CEACAM1/5/6/7), and metabolic enzymes (FABP1, CA4). Cluster 2 (17 genes) displayed an opposite pattern, with upregulation later in pseudotime. This cluster was enriched for goblet cell markers (MUC2, TFF3, FCGBP, CLCA1) and proliferation regulators (MKI67, TOP2A, CENPF), revealing a coordinated transcriptional program for stem cell to goblet cell differentiation.

The examination of the pseudotime-dependent expression of the three hub genes revealed distinct patterns (**Supplementary Figures 3C, D**). LCN2 was pronouncedly spatially specific and was highly expressed along the goblet cell differentiation path while being expressed at very low levels in stem cell-enriched regions. Quantitative analysis indicated that LCN2 displayed a distinct inverted U-shaped curve: low expression in early pseudotime (0–5), peak expression during mid-differentiation (5–12), and a decline in the late phase (12–20). This dynamic pattern was primarily driven by goblet cells, which showed the highest expression at the peak phase, and LCN2 became the only hub gene with a robust pseudotime-dependent change. APP showed a moderate decrease from early to late pseudotime with greater heterogeneity, whereas TNFSF4 maintained consistently low expression throughout the pseudotime, consistent with its T cell-specific expression pattern.

### 3.12 Cell-cell communication networks mediated by hub genes

To systematically dissect the intercellular communication landscape coordinated by hub genes in IBD, a comprehensive cell-cell interaction analysis was conducted using CellChat. Cells were stratified based on the expression status of hub genes (expression > 0 considered positive), generating subgroups for direct comparison of signaling capacity between gene-expressing and non-expressing populations.

For APP-mediated signaling, the analysis revealed that goblet cells and stem cells were the primary signal sources, with the APP(+) population exhibiting enhanced outgoing signal strength (**Supplementary Figures 4A, B**). This suggested that APP expression marks a functionally specialized epithelial subset, a critical hub for coordinating epithelial-immune crosstalk in the inflamed intestinal microenvironment. APP(+) goblet cells showed elevated signaling activity, particularly targeting immune cell populations including B cells, T cells, and plasma cells, as well as epithelial lineages such as DCLK1^+^ progenitors and Paneth cells. This indicated that APP-expressing goblet cells orchestrate both immune modulation and epithelial homeostasis. A heatmap identified APP along with COLLAGEN, LAMININ, FN1, and SEMA4 as prominent communication pathways (**Supplementary Figure 4C**). The analysis demonstrated that APP(+) goblet cells and stem cells exhibited increased outgoing signaling activity across multiple pathways. In contrast, DCLK1^+^ progenitors, MKI67^+^ proliferating cells, and Paneth cells emerged as primary signal receivers, suggesting that APP-mediated signaling might regulate epithelial regeneration and stem cell niche maintenance during intestinal inflammation. The ligand-receptor pair analysis identified key molecular interactions, including THBS1-SDC1, THBS1-integrin complexes (ITGA5 or ITGB1), SEMA4D-PLXNB1, and extensive laminin-integrin interactions (LAMC1, LAMB2, or LAMB3 with ITGA6, ITGB1, or ITGB4) (**Supplementary Figure 4D**). As shown in **Supplementary Figure 4D**, the APP(+) goblet cells primarily signaled through extracellular matrix pathways such as COLLAGEN and LAMININ, suggesting a potential role in driving niche remodeling and tissue repair during IBD pathogenesis. Moreover, collagen-integrin pairs showed significant communication probability that highlights the remodeling of extracellular matrices within APP-mediated signaling. These findings position APP as a key regulator of tissue architecture and cellular microenvironment in IBD. **Supplementary Figure 4E** further illustrates the cell communication network for the APP signaling pathway.

As a complement to APP signaling, LCN2 expression defined a functionally distinct goblet cell subpopulation with specialized communication capabilities. Goblet_cells_LCN2(+) showed enhanced outgoing signal strength compared to their LCN2(-) counterparts (**Supplementary Figures 5A, B**). This indicated that LCN2 expression was associated with a hyperactive secretory phenotype reflecting an adaptive response to OS and microbial challenges in IBD. The analysis of signaling patterns revealed that while goblet_cells_LCN2(+) exhibited elevated outgoing patterns, B cells, T cells, and plasma cells primarily acted as signal receivers, and stem cells demonstrated bidirectional communication capability (**Supplementary Figure 5C**). This suggested that LCN2-expressing goblet cells served as key modulators for adaptive immune responses while maintaining reciprocal interactions with the stem cell compartment to coordinate immune activation with tissue regeneration. In the context of the APP signaling pathway, goblet_cells_LCN2(-) and stem_cells_LCN2(-) primarily targeted B cells, DCLK1^+^ progenitors, MKI67^+^ proliferating cells, Paneth cells, plasma cells, and T cells (**Supplementary Figure 5D**). Notably, MKI67^+^ cells received strong signals from multiple LCN2-expressing populations, indicating that the proliferative activity was potentially regulated and that LCN2 might be a regulator in controlling the proliferation and regeneration of the intestinal epithelium during intestinal inflammation. **Supplementary Figure 5E** further presents the cell communication network for the APP signaling pathway.

In contrast to the epithelium-centric communication patterns of APP and LCN2, TNFSF4 expression in T cells conferred a distinct signaling profile. The analysis revealed that TNFSF4(+) T cells exhibited significantly higher numbers of interactions and stronger interaction weights compared with TNFSF4(-) T cells (**Supplementary Figures 6A, B**). This identified TNFSF4 expression as a marker for activated T cells with amplified signaling potential. TNFSF4(+) T cells demonstrated elevated outgoing signaling across multiple pathways while maintaining comparable incoming characteristics (**Supplementary Figure 6C**). This suggested that TNFSF4(+) T cells functioned primarily as signal transmitters, positioning them as key effector cells that modulate the behavior of epithelial cells during IBD pathogenesis. The ligand-receptor analysis identified specific communication axes with epithelial populations, particularly Paneth cells, DCLK1^+^ progenitors, and stem cells. This indicated that TNFSF4-mediated T cell signaling might influence critical functions of the intestine, including antimicrobial defense, epithelial differentiation, and tissue regeneration. As shown in **Supplementary Figure 6D**, the CD22 signaling pathway network indicated that TNFSF4(+) T cells preferentially targeted Paneth cells through the CD22 signaling pathway. This revealed a potential mechanism by which activated T cells modulated the secretion of antimicrobial peptides and the maintenance of the stem cell niche. **Supplementary Figure 6E** further illustrates the cell communication network for the CD22 signaling pathway.

Collectively, these findings revealed that hub genes orchestrated complementary communication networks. Specifically, APP and LCN2 primarily mediated epithelium-to-immune signaling via goblet cells and stem cells, whereas TNFSF4 enhanced immune-to-epithelium communication through T cells. This functional division of labor among the three hub genes reflects the multifaceted nature of OS responses in IBD, necessitating coordinated regulation at both epithelium and immune levels.

### 3.13 Virtual knockout simulation revealing the regulation networks of hub genes

To investigate the regulatory roles of the hub genes in UC, a virtual gene knockout analysis was performed on the GSE150115 single-cell dataset (1,091 cells, 3,000 highly variable genes) using ‘scTenifoldKnk’. This method constructs gene regulation networks through tensor decomposition and compares network perturbations before and after virtual knockout.

For the APP knockout, the differential regulation analysis identified APP itself as the most significantly altered gene, followed by SSR4 and MZB1 (**Supplementary Figure 7A**). A volcano plot demonstrated that genes with |Z-score| > 2 and p.adj < 0.05 were primarily enriched for extracellular matrix organization (SPARC, LAMC1, LAMB1) and immune regulation pathways (IGFBP7, A2M), suggesting that APP was critical in the maintenance of intestinal epithelial barriers and the regulation of inflammatory responses (**Supplementary Figure 7B**).

Similarly, the LCN2 knockout identified LCN2 as the most differentially regulated gene, with SSR4 and MZB1 also significantly affected (**Supplementary Figure 7C**). Notably, the regulatory changes of genes involved in epithelial-mesenchymal transition (S100A10, KRT18, EPCAM) and cell adhesion (ANXA2, CEACAM5) were significant, indicating that LCN2 might participate in the integrity of intestinal epithelium and the stress response of cells (**Supplementary Figure 7D**).

TNFSF4 was excluded from the in silico knockout analysis due to its expression being confined to T cells with minimal detection in other cell types, resulting in insufficient data after quality control filtering. This technical limitation aligns with the cell-cell communication analysis, which showed a specialized role for TNFSF4 in T cell-mediated immune-to-epithelium signaling rather than broad epithelial functions. These findings highlighted that APP and LCN2 served as key regulatory nodes in coordinating OS responses and inflammation processes in the IBD microenvironment.

### 3.14 Experimental verification of hub genes in a murine model of DSS-triggered colitis

**Figure 7:**
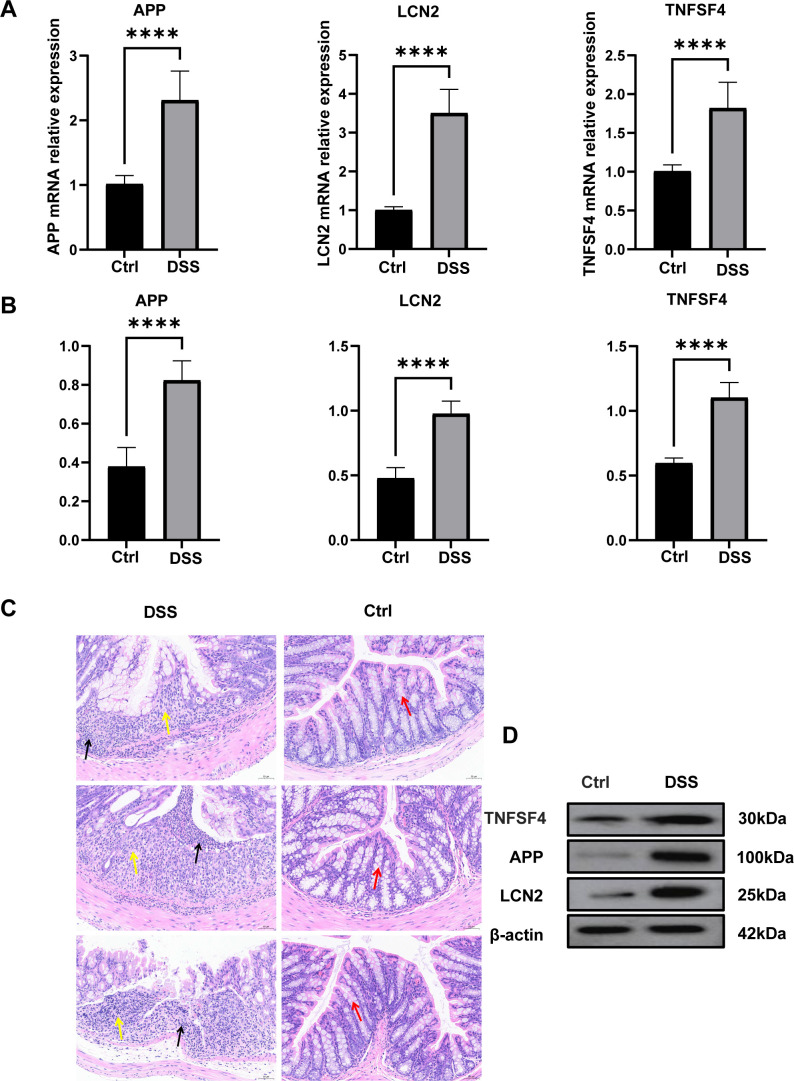
Experimental validation of hub genes in a murine model of DSS-triggered colitis. **(A)** RT-qPCR validation. Bar plots show the relative expression levels of mRNA of LCN2, APP, and TNFSF4 in colon tissues of the DSS-treated mice and the control group. Compared with the control group, the expression of all three hub genes was significantly elevated in the DSS group. ****P < 0.0001. (B&D) Western blotting validation. **(B)** Greyscale value quantification of Western blotting bands. Bar plots display the relative protein expression levels of TNFSF4, APP, and LCN2 normalized to β-actin. Compared with the control group, the protein expression of all 3 hub genes was markedly heightened in the DSS group. ****P < 0.0001. **(C)** Histopathological examination of colon tissues by H&E staining (20×magnification). **(D)** Western blotting band images: the protein expression of TNFSF4, APP, and LCN2 in colon tissues of control and DSS-administered mice, with β-actin utilized as the loading control.

To validate the expression patterns of the identified hub genes in a murine model of DSS-induced colitis, such a model was established, and histopathology, RT-qPCR, and WB analyses on colon tissues were performed. Successful establishment of the DSS-induced colitis model was confirmed through histopathological examination of colonic sections via H&E staining (**Figure 7C**). In the DSS model group, the overall structure of the colonic tissue was abnormal. Extensive diffuse erosion could be seen in the mucosal layer of the tissue, and the normal structure of the mucosal layer basically disappeared. The intestinal glands of the tissue were largely damaged, and goblet cells were basically not visible. These abnormal morphological features are indicated by the yellow arrows in **Figure 7C**. Pronounced infiltration of inflammatory cells was observed throughout the entire mucosal layer, as shown by the black arrows in **Figure 7C**. In contrast, the overall architecture of the colonic tissue in the control group remained largely intact. The cells in the tissue mucosal layer were arranged regularly. The columnar epithelium of the tissue mucosal layer was arranged in a tubular shape, and there were a large number of goblet cells sandwiched between the epithelial cells. These normal morphological features are indicated by the red arrows in **Figure 7C**. No significant loosening, edema, or necrosis was observed in the submucosa of the tissues, and no pronounced infiltration of inflammatory cells was noted in the tissues.

Building upon this histopathological validation, the expression of the hub genes was subsequently examined. Consistent with our bioinformatic predictions, the RT-qPCR analysis revealed that the mRNA expression levels of all 3 hub genes, comprising LCN2, APP, and TNFSF4, were significantly elevated in the DSS group compared with the control group (P <0.0001) (**Figure 7A**). These findings were additionally confirmed at a protein level via WB analysis. In comparison with the control group, the protein expression of TNFSF4, APP, and LCN2 was considerably upregulated in colon tissues of DSS-treated mice (all P <0.0001) (**Figures 7B, D**). These results have provided strong experimental evidence supporting the reliability of our bioinformatic analysis and have suggested that LCN2, APP, and TNFSF4 are critical in IBD pathogenesis through OS-related mechanisms.

## 4 Discussion

This study, employing an integrated multi-omics approach combining DEGs analysis, WGCNA, and dual ML algorithms (LASSO regression and RF), has systematically confirmed and deepened the understanding of the specific roles of three OS-related genes (LCN2, APP, and TNFSF4) in IBD. The novelty of this research is fourfold: (1) the integration of WGCNA with dual ML algorithms significantly reduced algorithmic bias compared with single-method approaches; (2) a three-tiered filtering system spanning DEGs, WGCNA modules, and the OS gene repository ensured biological relevance; (3) a comprehensive multi-tiered validation framework (three independent external cohorts, scRNA-seq, immune infiltration analysis, and DSS model) provided a complete chain of evidence; and (4) advanced analyses, including single-cell analysis, CellChat-based cell-cell communication mapping, and scTenifoldKnk virtual knockout simulations, systematically revealed, for the first time, the specific mechanisms by which these genes mediate OS-immunity-inflammation interactions in IBD pathogenesis.

The diagnostic performance of LCN2 for IBD was robust (AUC = 0.781), consistent with the established role of LCN2 as an acute-phase protein in inflammatory responses. Primarily produced by activated neutrophils and intestinal epithelial cells, LCN2 participates in host defense by regulating iron homeostasis and enhancing bacterial clearance in macrophages (36). The findings in this study provide multiple lines of evidence supporting the central role of LCN2 in OS-driven inflammation. First, immune infiltration analysis revealed robust positive associations between LCN2 expression and the infiltration of monocytes, M0 macrophages, and neutrophils. This pattern directly corresponds to the significant enrichment of NET formation pathways identified by GSEA. In IBD pathogenesis, excessive neutrophil infiltration and NET formation represent key mechanisms that aggravate OS, and inflammation-activated neutrophils exacerbate oxidative damage through massive ROS production and release of oxidases such as myeloperoxidase (37, 38). The single-cell analysis further revealed high LCN2 expression in goblet cells and intestinal stem cells, suggesting that LCN2 was involved in maintaining intestinal barrier function and tissue repair. This cell localization functionally aligns with the enrichment of antioxidant pathways, including glutathione metabolism and ROS detoxification. The regulation of LCN2 in intestinal OS involves complex interactions among NF-κB, Nrf2, and MAPK signaling pathways. In DSS-induced colitis models, LCN2 expression in intestinal epithelial cells is driven by MyD88-mediated NF-κB activation. MyD88 knockout mice show 60% reduced LCN2 levels with exacerbated inflammation (40% increase in DAI scores) (39). Conversely, the transcription factor NFE2L3 suppresses LCN2 expression by binding to MARE elements in the LCN2 promoter. NFE2L3 knockout mice exhibit 2.3-fold elevation in LCN2 mRNA after DSS treatment, accompanied by attenuated oxidative injury (30% reduction in malondialdehyde levels) (40). The MAPK pathway also modulates LCN2 expression: p38 MAPK phosphorylation enhances IκBζ transcriptional activity to upregulate LCN2, while the p38 inhibitor SB203580 reduces LCN2 expression by 50% among DSS-treated mice (41).

In addition to transcriptional regulation, OS directly induces LCN2 expression. H_2_O_2_ treatment increases LCN2 mRNA levels in the colonic epithelium three−fold, and this effect is further amplified to five-fold by the Nrf2 activator tBHQ (41). Supporting the clinical relevance of these mechanisms, LCN2 expression in the colonic mucosa of patients with IBD is positively associated with the OS marker 8-OHdG (r=0.72, p<0.001) (42). The regulation network of LCN2 extends to the gut microbiota. In LCN2-deficient mice, intestinal Enterobacteriaceae abundance increases two-fold, and secreted LPS activates the NF-κB pathway through TLR4 to feedback-induced LCN2 expression (39). Conversely, probiotics such as *Akkermansia muciniphila* suppress excessive LCN2 expression by producing short-chain fatty acids such as butyrate: in chronic colitis models, *Akkermansia muciniphila* treatment reduces LCN2 levels in feces by 35% (43). These findings establish LCN2 as a critical mediator linking gut microbiota dysbiosis, OS, and immune activation in IBD.

The diagnostic performance of APP was the highest (AUC = 0.805). Although APP is well characterized in neurodegenerative diseases, its role in IBD-associated OS remains poorly elucidated (44). In DSS-induced colitis models, APP mRNA levels in the intestinal mucosa are significantly elevated, and APP upregulation in colonic epithelial cells has been associated with intestinal inflammation (45, 46). These observations suggest a potential association between APP and IBD pathogenesis that needs to be mechanistically investigated. Current evidence indicates that APP may influence intestinal OS through three distinct mechanisms. First, the APP intracellular domain (AICD) interacts with the PIKfyve complex to regulate PI (3, 5)P_2_ metabolism, thereby modulating lysosomal function and cell OS levels (47). Second, aberrant APP processing generates pathogenic products: Aβ peptides catalyze ROS production by binding metal ions (Cu²^+^ and Fe³^+^), while the C99 fragment impairs lysosomal function and reduces ROS clearance capacity within cells (48, 49). Third, APP may indirectly affect OS via gut microbiota alterations. APP NL-G-F mice exhibit increased Bacteroidetes/Firmicutes ratio, a dysbiosis pattern associated with elevated intestinal OS (50). The immune infiltration analysis revealed distinct correlation patterns: APP expression was positively associated with monocytes, M0 macrophages, and plasma cells, but was negatively associated with NK cells and T-cell subsets. This bidirectional pattern suggests that APP may differentially regulate innate versus adaptive immune responses. Supporting this interpretation, GSEA identified significant enrichment of PI3K-Akt and NF-κB signaling pathways, both of which are central to immune cell activation and OS regulation. Previous studies have shown that APP regulates macrophage polarization and the capability of producing ROS, suggesting a potential mechanistic link between APP expression and the observed immune infiltration patterns (46). Despite these findings, the precise role of APP in IBD pathogenesis remains incompletely defined. Since research on the intestinal functions of APP, combined with the complexity of its interactions with OS, immunity, and microbiota, is limited, additional mechanism studies are needed to establish causal relationships.

TNFSF4, a member of the TNF superfamily, exhibited favorable diagnostic performance (AUC = 0.754). Although TNFSF4 is well established as a T-cell co-stimulatory molecule, its involvement in IBD-associated OS remains incompletely clarified. Existing studies have revealed that TNFSF4 facilitates the activation, proliferation, and survival of T-cells through interaction with its receptor OX40, and targeting the OX40/OX40L pathway has been proven to be effective in chronic colitis models (51, 52). These observations suggest a potential link between TNFSF4-mediated immune activation and OS in IBD. The relationship between TNFSF4 and OS appears to be bidirectional. OS promotes TNFSF4 expression through epigenetic modifications and transcription factor activation (53). Moreover, TNFSF4 reciprocally modulates OS levels by regulating Nrf2 and NF-κB signaling pathways (54). This bidirectional interaction suggests that TNFSF4 may function as both a responder to and amplifier of OS in the inflammatory microenvironment. The immune infiltration analysis revealed that TNFSF4 expression was positively associated with monocytes, M0 macrophages, and neutrophils. This pattern is consistent with GSEA-identified enrichment of IL-17 signaling pathway, cytokine-cytokine receptor interaction, and chemokine signaling pathway, all central to inflammatory responses in IBD. The single-cell analysis further localized TNFSF4 expression predominantly to T cells, aligning with its established function as a T-cell co-stimulatory molecule. These findings suggest that TNFSF4-mediated T-cell activation contributes to sustained inflammatory responses and subsequent OS amplification through recruitment and activation of innate immune cells. In addition to immune regulation, environmental factors modulate the TNFSF4-OS axis. A high-fat diet simultaneously increases intestinal OS and TNFSF4 expression (55), while probiotics supplementation reduces both parameters (56). These observations indicate that gut microbiota composition and dietary patterns contribute to the regulation network linking TNFSF4, OS, and inflammation. Nevertheless, the multifactorial nature of these interactions needs to be further mechanistically studied to clarify the causal relationships governing the role of TNFSF4 in IBD pathogenesis.

To validate the diagnostic utility of these three hub genes, external validation was performed across three independent IBD cohorts covering 618 samples (**Supplementary Table 2**). Of note, the diagnostic performance of LCN2 varied by platform: microarray-based cohorts yielded AUC values of 0.781, 0.953, and 0.844, whereas the RNA-seq cohort (GSE165512) showed a substantially lower AUC of 0.643. This pattern indicated that technical platform differences, rather than disease phenotype composition, might constitute the primary driver of LCN2 performance variability. Supporting this interpretation, the proportion of UC and CD patients across cohorts (UC: 24.7%-56.4%; CD: 43.6%-75.3%) showed no pronounced correlation with the diagnostic performance of LCN2. Notably, detailed clinical metadata regarding sample collection sites (inflamed mucosa vs. non-inflamed mucosa) and disease activity status were not fully documented in the original databases, and these factors may also influence diagnostic performance.

In contrast to the platform-dependent performance of LCN2, APP and TNFSF4 demonstrated relatively consistent diagnostic values across all validation cohorts (APP AUC range: 0.606-0.677; TNFSF4 AUC range: 0.632-0.686). However, the performance of both genes was reduced compared with the discovery cohort (APP: 0.805-0.606; TNFSF4: 0.754 - 0.632). This observation reflected the complex multifactorial regulation of APP and TNFSF4 expression. Unlike LCN2, primarily produced by neutrophils with relatively straightforward regulatory mechanisms, APP and TNFSF4 were regulated by multiple signaling pathways and influenced by diverse immune cell infiltration patterns, sample collection sites, and disease activity status. The heterogeneity in these factors across validation cohorts possibly contributed to increased expression heterogeneity of APP and TNFSF4, thereby explaining their attenuated diagnostic performance in external validation. Nevertheless, both genes maintained moderate diagnostic value across all validation cohorts, supporting their utility as complementary biomarkers to enhance diagnostic specificity when used in combination with LCN2.

The synergistic actions of the 3 hub genes form a complex network regulating OS in IBD. The functional enrichment analyses revealed significant activation of multiple OS-related pathways, including glutathione metabolism and the metabolic process of ROS. The activation of these pathways, alongside the observed elevation in innate immune cells and reduction in adaptive immune cells in immune infiltration analysis, forms a complete pathological response. A study has shown that modulation of glutathione levels influences the production of pro-inflammatory factors and ROS (57). Furthermore, superoxide dismutase 2 (SOD2) maintains intracellular oxidative balance through the scavenging of ROS, and its deficiency triggers the accumulation of ROS, thereby suppressing the innate immune responses mediated by RIG-I-like receptors (58). These findings highlight that the metabolism of ROS and glutathione was crucial for immune regulation and OS.

Based on these mechanistic insights, the three hub genes demonstrate significant clinical potential. First, as early diagnostic markers, elevated expression of these three genes before IBD symptom onset could serve as early warning indicators. Second, for dynamic assessment of disease activity, our immune infiltration analysis revealed that the expression of these three genes correlates closely with M0 macrophages, monocytes, and neutrophils (p<0.01)—key cellular indicators of IBD inflammatory activity. Monitoring these gene expression levels may therefore provide real-time insights into disease status and guide therapeutic intensity adjustments. Third, regarding the prediction of anti-inflammatory treatment response, since all three genes are enriched in critical inflammatory signaling pathways including NF-κB and PI3K-Akt, their expression changes may predict patient responsiveness to anti-inflammatory therapy, though prospective clinical studies are needed for validation.

Furthermore, the expression patterns of these three genes provide a theoretical foundation for individualized treatment strategies. For targeted antioxidant therapy, patients with high LCN2 and APP expression exhibit elevated intestinal OS burden. Based on this mechanism, antioxidant agents (such as N-acetylcysteine and selenium supplementation) may offer more targeted therapeutic benefits for LCN2-high patients, though clinical cohort studies are required to validate actual efficacy. For immunomodulatory therapy, TNFSF4’s specific expression on T cells and elevated levels suggest Th17/Treg imbalance. These findings suggest that patients with high TNFSF4 expression may benefit from biologics targeting the OX40/OX40L pathway (such as OX40 monoclonal antibodies), warranting exploration in clinical trials. Regarding combined therapeutic strategies, patients with elevated APP and LCN2 expression demonstrate enhanced epithelial-immune cell bidirectional communication, indicating the need for simultaneous epithelial barrier protection and immune modulation. Such patients may benefit from combined application of probiotic formulations (to modulate gut microbiota and attenuate LCN2 overexpression) and biologics (to regulate excessive immune responses), though specific strain selection and formulation combinations require further clinical validation.

Nevertheless, several limitations of this study warrant acknowledgment. First, the relatively low log_2_FC threshold (0.5) employed for initial DEG screening may have retained genes with modest expression alterations. However, the subsequent stringent filtering through WGCNA, ML algorithms, and external validation effectively mitigated this potential bias. Second, the analyses were primarily based on limited public datasets, and validation in larger, multi-center patient cohorts is warranted to corroborate the universal applicability of our findings. Third, while disease subtypes (UC vs. CD) were documented across all cohorts (**Supplementary Table 2**), detailed clinical phenotyping, including disease activity scores, endoscopic severity grades, and treatment history, was not comprehensively available in these public repositories. This may contribute to the observed biomarker performance variability, reflecting both technical platform differences and uncontrolled clinical factors. Nevertheless, the consistent performance across cohorts with varying UC/CD ratios (24.7%-56.4% UC) suggests robust disease-associated signals, though future prospective studies with standardized clinical phenotyping are needed to validate clinical utility and establish relationships with disease activity. Fourth, although key OS-related biomarkers were identified through multiple bioinformatics approaches, the exact mechanisms of these three hub genes in IBD pathogenesis require further elucidation. Specifically, the virtual knockout analysis (scTenifoldKnk) predicted transcriptional network changes based on correlation patterns and failed to account for protein-protein interactions or post-translational modifications. Thus, its results should be interpreted as hypothesis-generating rather than definitive causal relationships. In the future, loss-of-function experiments using siRNA or CRISPR/Cas9 technology in intestinal organoids or cell lines are warranted to validate the predicted effects on target genes such as SPARC, LAMC1, and S100A10, and to assess their impacts on barrier integrity, inflammatory responses, and OS.

## 5 Conclusion

In summary, in this study, 3 hub genes (LCN2, APP, and TNFSF4) closely associated with OS in IBD have been successfully identified by innovatively integrating multiple bioinformatics approaches. Through a systematic analysis of the expression patterns, immune infiltration correlations, and functional pathway enrichment of these 3 genes, a complex interaction network involving OS, immune imbalance, and inflammatory responses was revealed. In addition to deepening the understanding of IBD pathogenesis, these findings lay a vital molecular foundation for a precise diagnosis and an individualized treatment of IBD.

## Data Availability

The datasets presented in this study can be found in online repositories. The names of the repository/repositories and accession number(s) can be found in the article/**Supplementary Material**.
