# Supplementary material for: Identification of oxidative stress-associated biomarkers for inflammatory bowel disease through integrated machine learning and weighted gene co-expression network analysis
Source: Front Immunol. 2026 Jun 10;17:1763786. doi: 10.3389/fimmu.2026.1763786 (PMC13291112; doi:10.3389/fimmu.2026.1763786)
Supplement: Supplementary file 1 [file Table1.docx]

**Table S1. Datasets and Their Features**

| Dataset | Database | Platform | Data Type | Sample |
| --- | --- | --- | --- | --- |
| GSE3365 | GEO | GPL96 | Microarray | 85 patients with IBD and 42 controls |
| OS-related genes | GeneCards | GeneCards | GeneCards | Obtained OS-related genes from the GeneCards database |
| GSE150115 | GEO | GPL18573 | Single-cell | Tissue samples from 5 patients suffering from ulcerative colitis |
| IBD: inflammatory bowel disease; OS: oxidative stress; GEO: Gene Expression Omnibus | | | | |

**Table S2. Characteristics of Validation Cohorts**

| **Cohort** | **GSE3365**  **(Discovery)** | **GSE75214** | **GSE179285** | **GSE165512** |
| --- | --- | --- | --- | --- |
| Platform | GPL96 (Microarray) | GPL6244 (Microarray) | GPL6480 (Microarray) | GPL16791 (RNA-seq) |
| Total Samples | 127 | 194 | 254 | 170 |
| UC Samples | 26 | 97 | 55 | 40 |
| CD Samples | 59 | 75 | 168 | 84 |
| Control Samples | 42 | 22 | 31 | 46 |
| UC Proportion | 30.60% | 56.40% | 24.70% | 32.30% |
| CD Proportion | 69.40% | 43.60% | 75.30% | 67.70% |
| Disease Activity (UC) | NA | Active:74, Inactive:23 | NA | NA |
| Disease Activity (CD) | NA | Active:59, Inactive:16 | NA | NA |
| Inflammation Status (UC) | NA | NA | Inflamed:23, Non-inflamed:32 | NA |
| Inflammation Status (CD) | NA | NA | Inflamed:47, Non-inflamed:121 | NA |
| Sample Location (UC) | peripheral blood mononuclear cells | Colon:97 | Colon:55 | Colon:40 |
| Sample Location (CD) | peripheral blood mononuclear cells | Ileum:67, Colon:8 | Ileum:82, Colon:86 | Ileum:44, Colon:40 |
| Sample Location (Control) | peripheral blood mononuclear cells | Ileum:11, Colon:11 | Ileum:8, Colon:23 | Ileum:11, Colon:35 |
|  |  |  |  |  |
| Age Range (UC) | 25-73 years | NA | NA | NA |
| Age Range (CD) | 20-65 years | NA | NA | NA |
| Age Range (Control) | 25-60 years | NA | NA | NA |
| Sex Distribution (UC) | Male:8, Female:18 | NA | NA | NA |
| Sex Distribution (CD) | Male:21, Female:38 | NA | NA | NA |
| Sex Distribution (Control) | Male:24, Female:18 | NA | NA | NA |

**Table S3. Sequences of Primers for RT-qPCR**

| Gene | Forward Primer (5'→3') | Reverse Primer (5'→3') | Annealing  Temperature (℃) |
| --- | --- | --- | --- |
| GAPDH | CATCACTGCCACCCAGAAGACTG | ATGCCAGTGAGCTTCCCGTTCAG | 60 |
| TNFSF4 | GGAAGAAGACGCTAAGGCTGGT | CTGGTAACTGCTCCTCTGAGTC | 60 |
| APP | TCCGTGTGATCTACGAGCGCAT | GCCAAGACATCGTCGGAGTAGT | 60 |
| LCN2 | ATGTCACCTCCATCCTGGTCAG | GCCACTTGCACATTGTAGCTCTG | 60 |

| 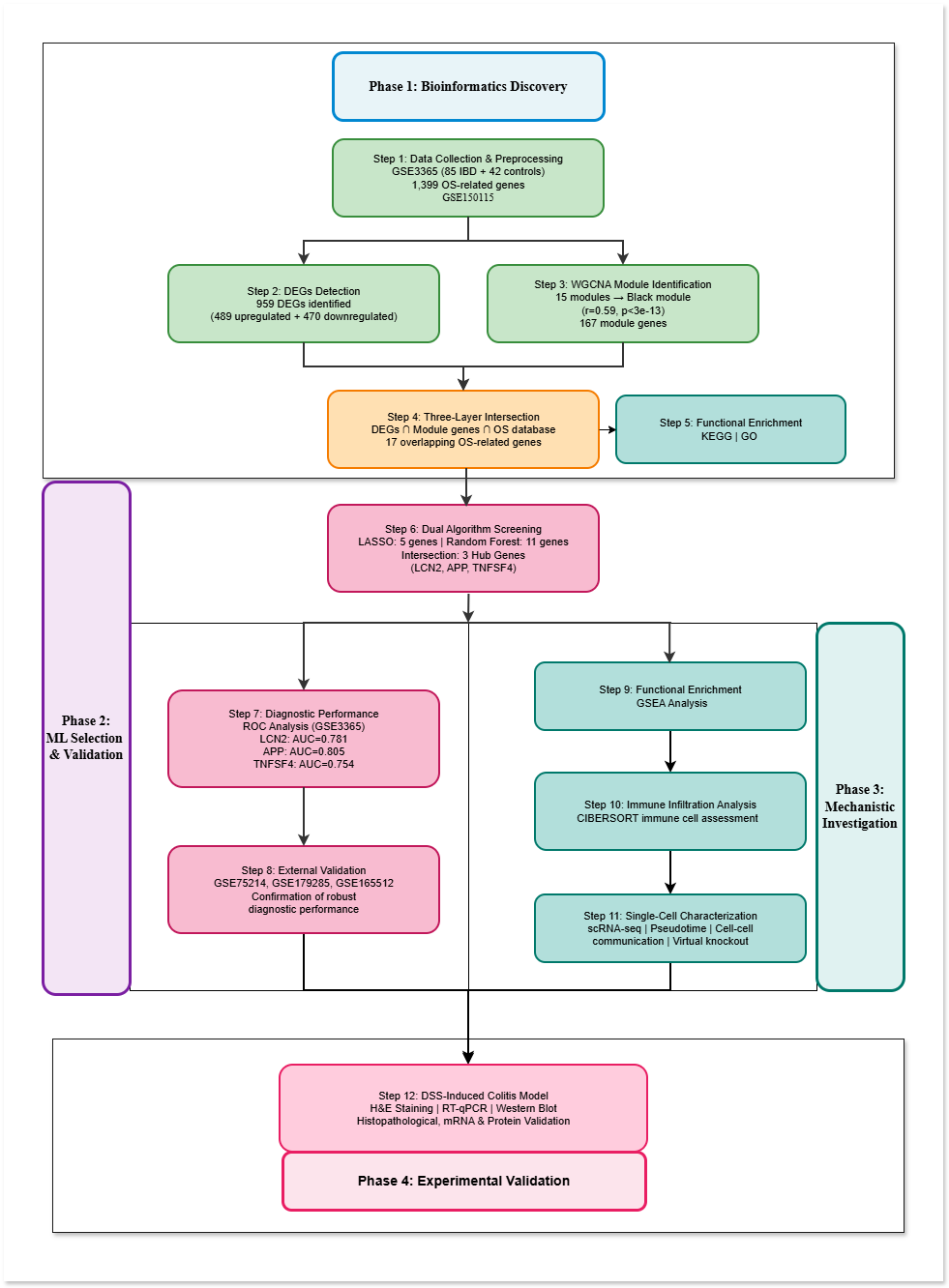 |
| --- |
| 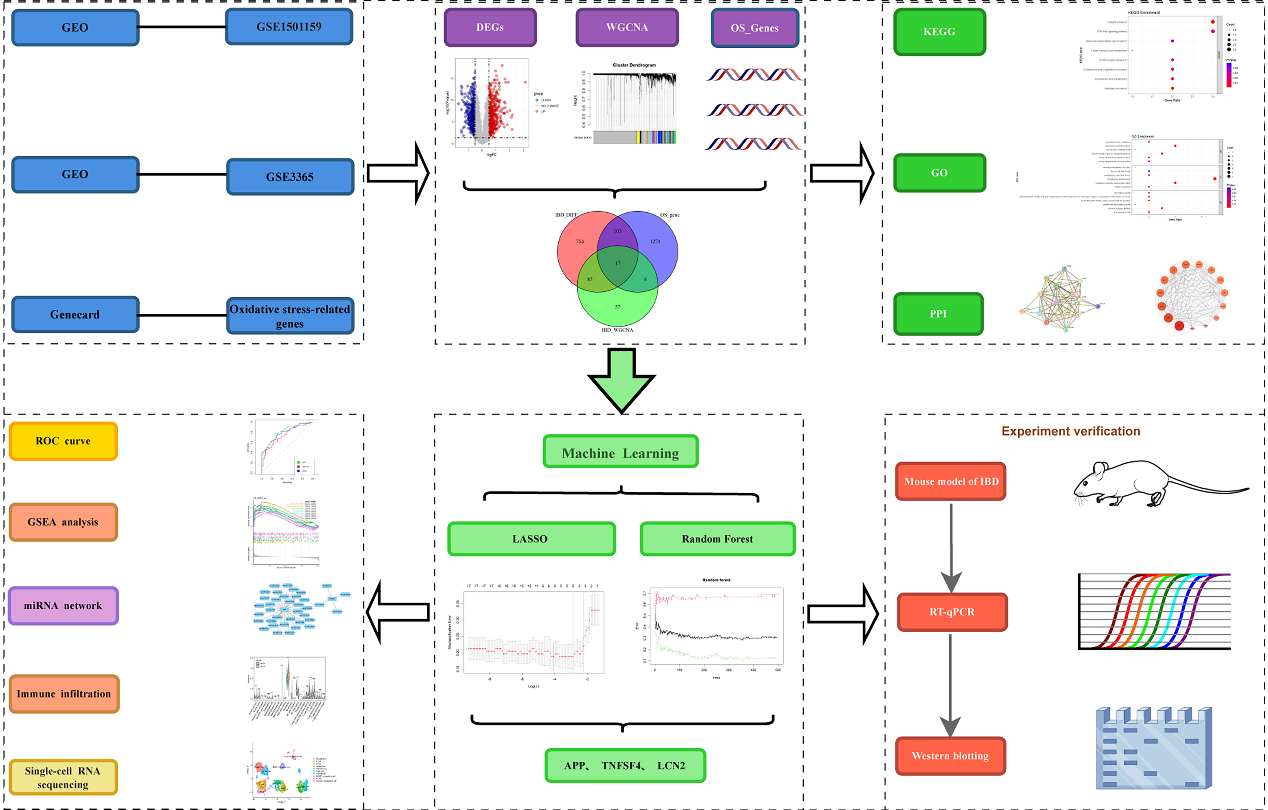 |
| **Figure S1.** Flowchart for Bioinformatic Design and Experimental Validation Process |


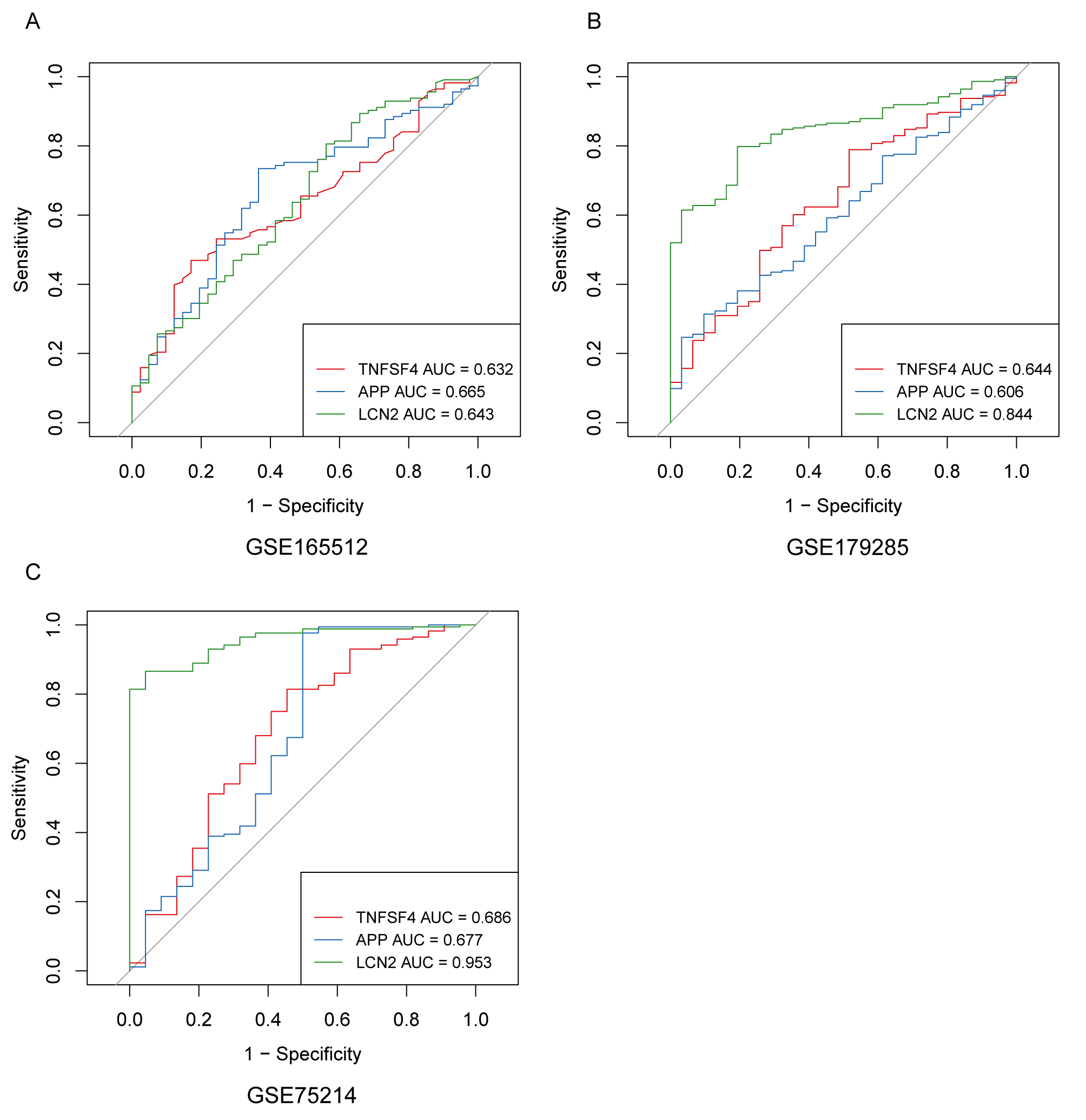


**Figure S2.** ROC curve for the 3 hub genes (APP, TNFSF4, and LCN2) in GSE165512(A), GSE179285(B), and GSE75214(C).


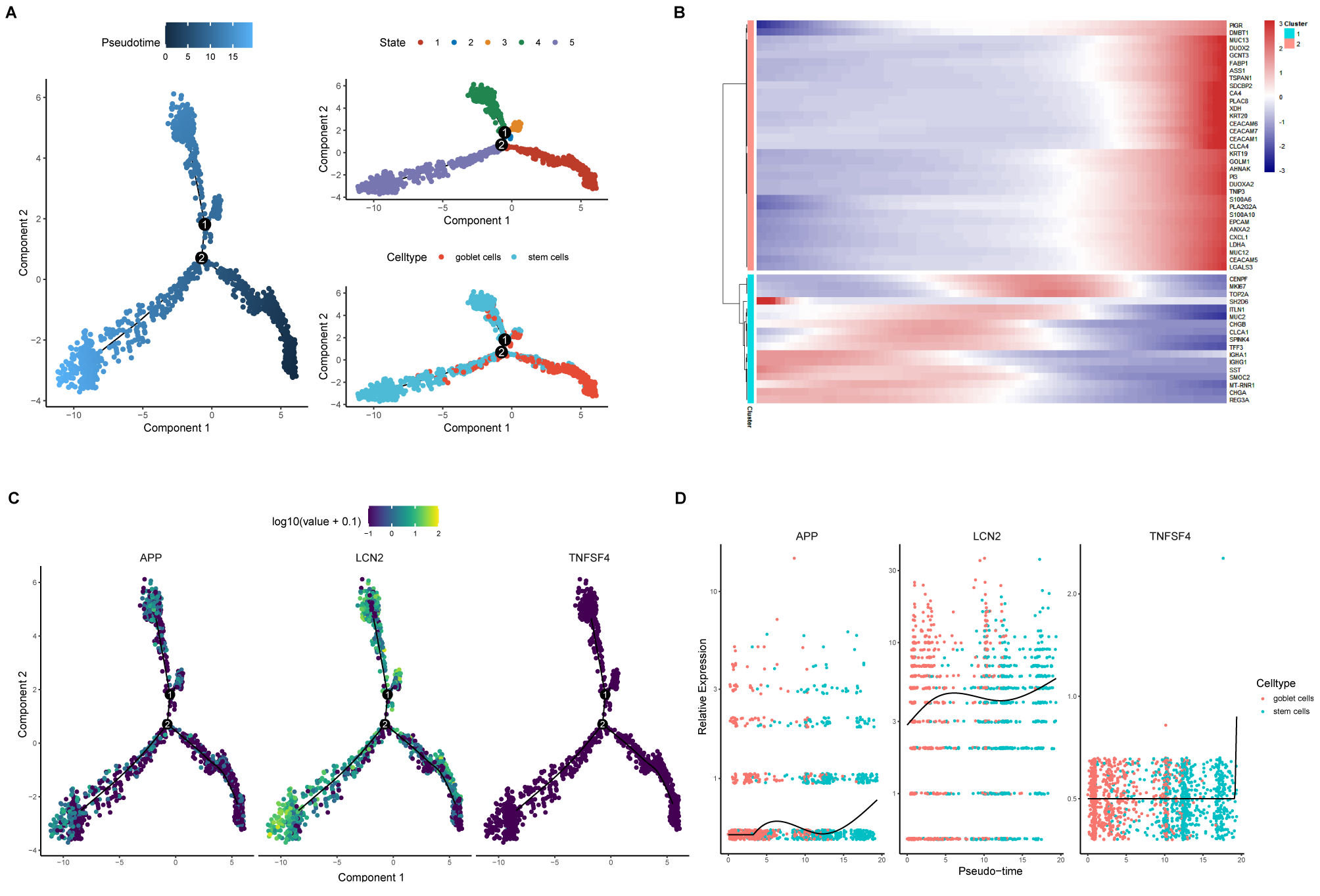


**Figure S3.** Developmental trajectory analysis of stem cells and goblet cells


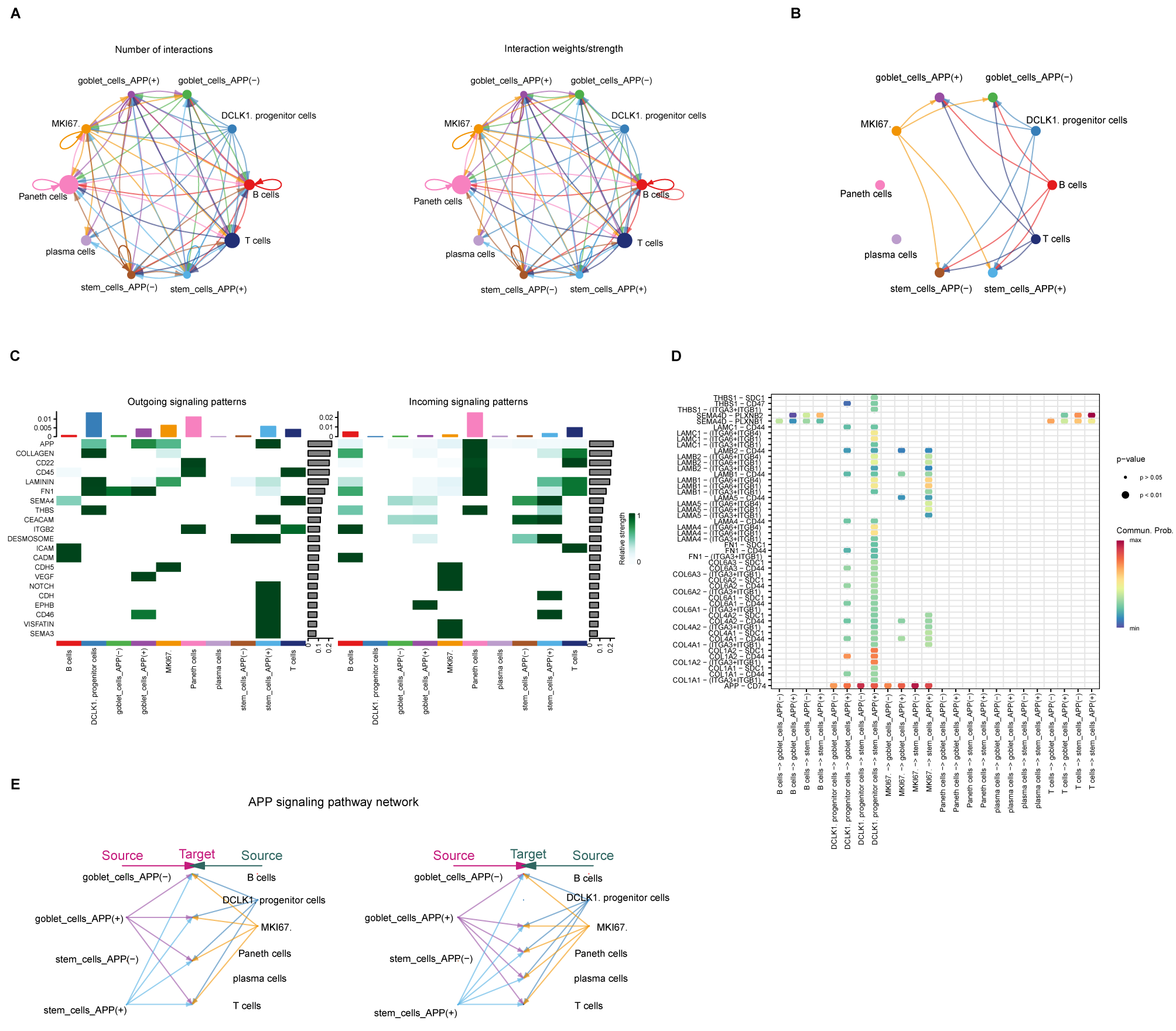


**Figure S4**. Expression of APP in stem or goblet cells and cell-cell communication


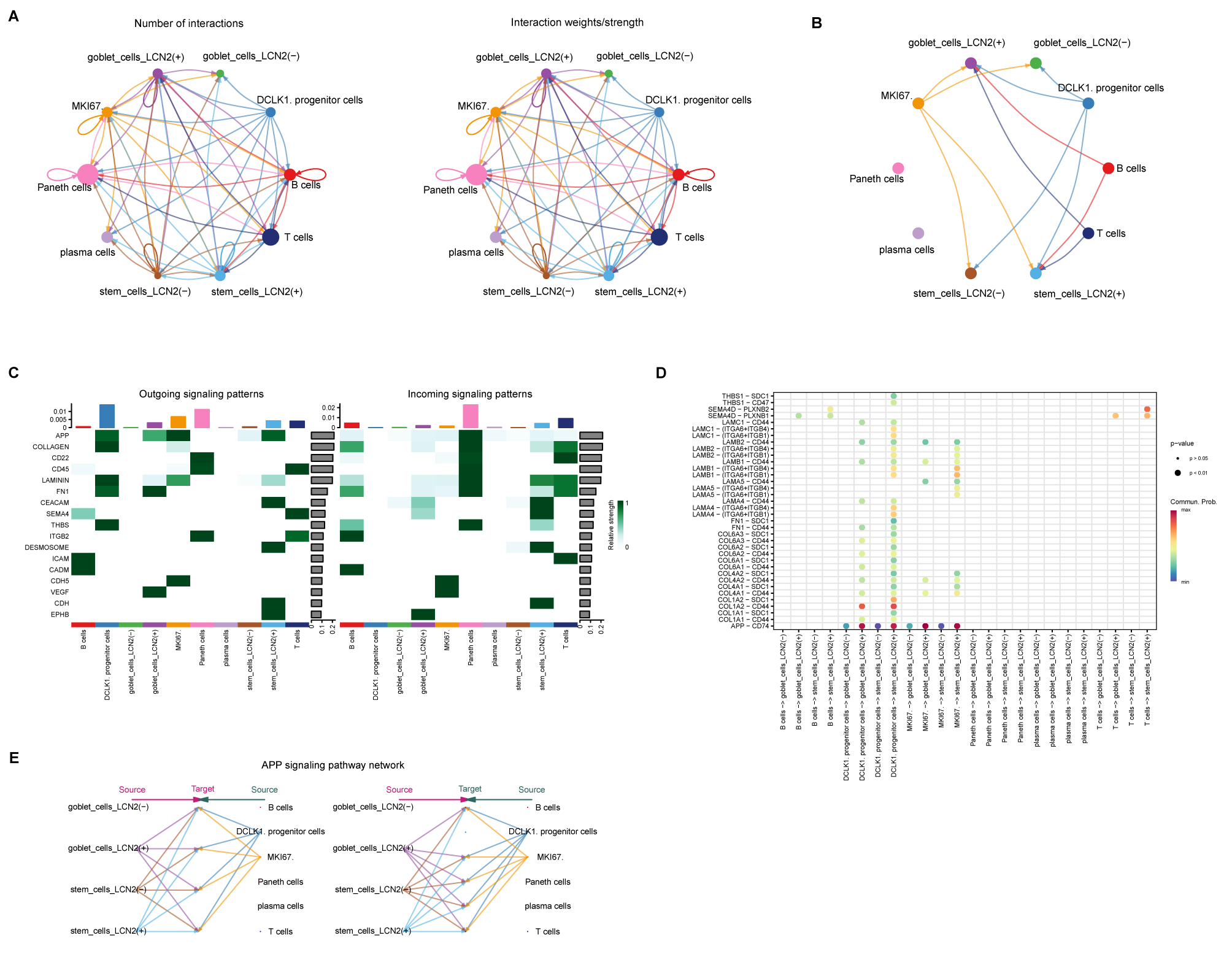


**Figure S5**. Expression of LCN2 in stem or goblet cells and cell-cell communication


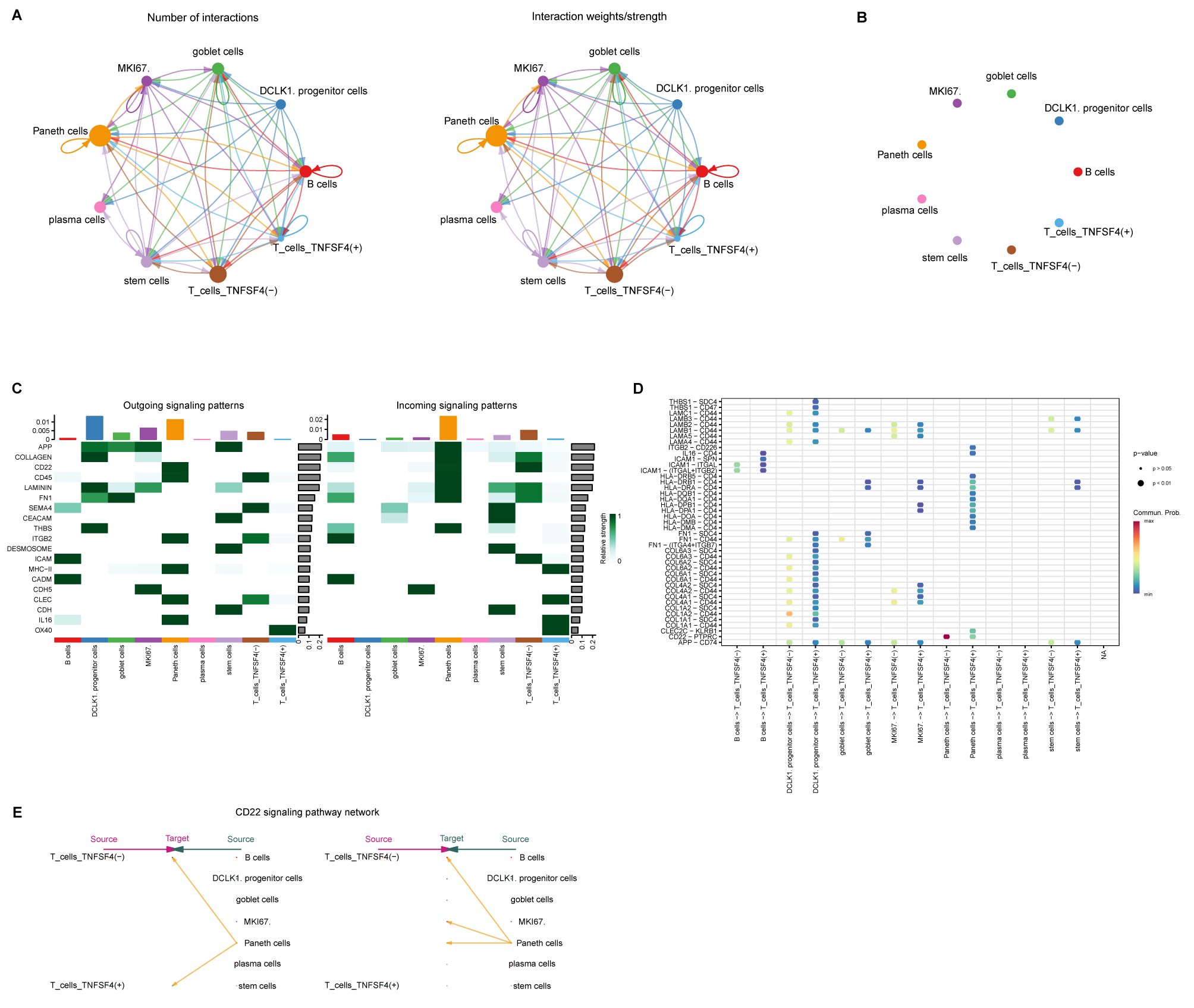


**Figure S6**. Expression of TNFSF4 in T cells and cell-cell communication


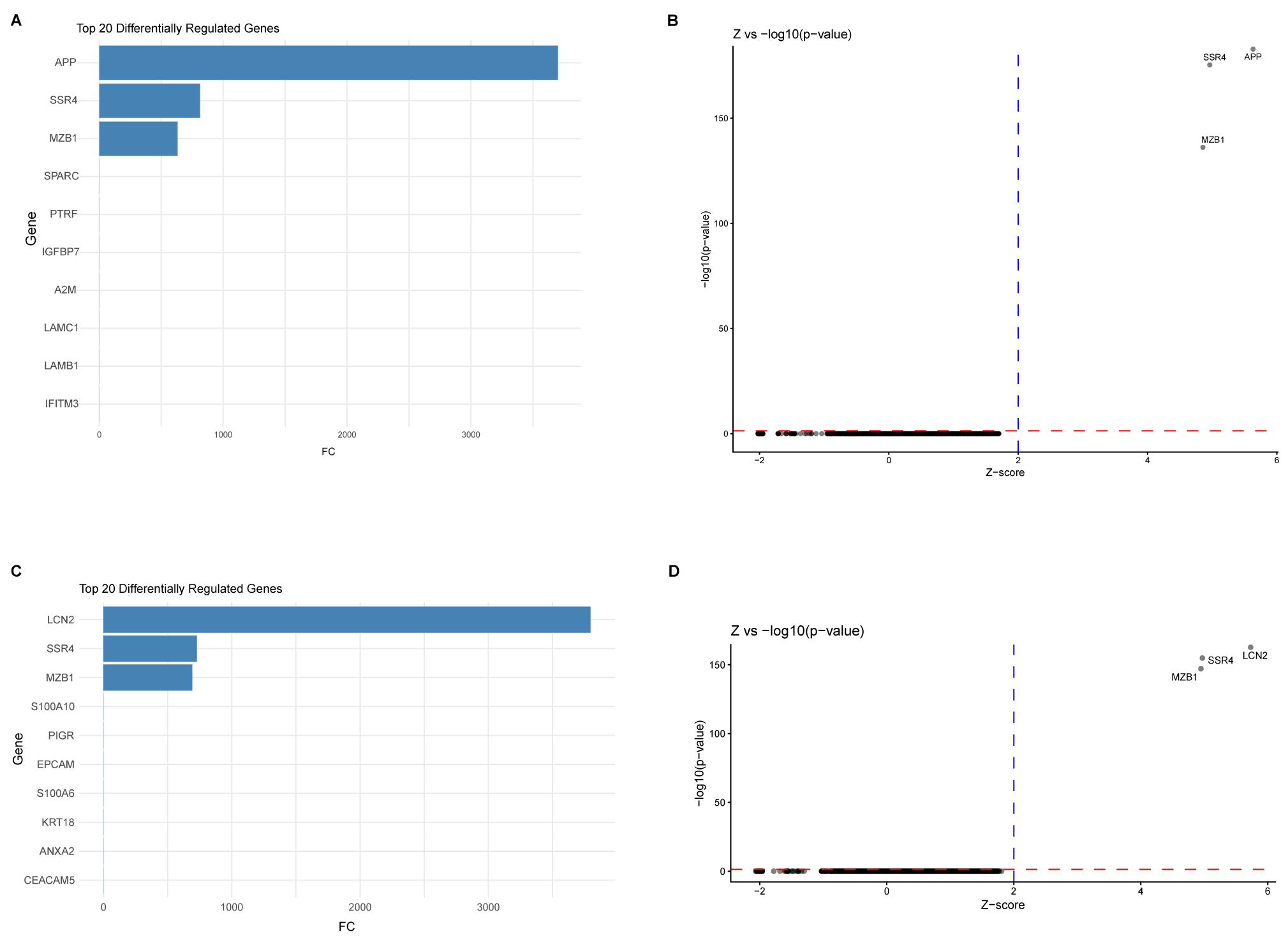


**Figure S7**. Virtual knockout analysis of APP and LCN2
